# Learning by Heart: Cultural Patterns in the Faunal Processing Sequence during the Middle Pleistocene

**DOI:** 10.1371/journal.pone.0055863

**Published:** 2013-02-20

**Authors:** Ruth Blasco, Jordi Rosell, Manuel Domínguez-Rodrigo, Sergi Lozano, Ignasi Pastó, David Riba, Manuel Vaquero, Josep Fernández Peris, Juan Luis Arsuaga, José María Bermúdez de Castro, Eudald Carbonell

**Affiliations:** 1 IPHES, Institut Català de Paleoecologia Humana i Evolució Social, Tarragona, Spain; 2 Àrea de Prehistòria, Universitat Rovira i Virgili (URV), Tarragona, Spain; 3 The Gibraltar Museum, Gibraltar; 4 Department of Prehistory, Complutense University, Madrid, Spain; 5 IDEA (Instituto de Evolución en África), Museo de los Orígenes, Madrid, Spain; 6 SIP (Servei d’Investigació Prehistòrica), Museo de Prehistoria, Diputación de Valencia, Valencia, Spain; 7 Departamento de Paleontología, Facultad de Ciencias Geológicas, Complutense University, Madrid, Spain; 8 Centro de Investigación (UCM-ISCIII) de Evolución y Comportamiento Humanos, Madrid, Spain; 9 CENIEH (Centro Nacional de Investigación sobre Evolución Humana), Burgos, Spain; 10 Institute of Vertebrate Paleontology and Paleoanthropology (IVPP), Beijing, China; University of Oxford, United Kingdom

## Abstract

Social learning, as an information acquisition process, enables intergenerational transmission and the stabilisation of cultural forms, generating and sustaining behavioural traditions within human groups. Archaeologically, such social processes might become observable by identifying repetitions in the record that result from the execution of standardised actions. From a zooarchaeological perspective, the processing and consumption of carcasses may be used to identify these types of phenomena at the sites. To investigate this idea, several faunal assemblages from Bolomor Cave (Valencia, Spain, MIS 9-5e) and Gran Dolina TD10-1 (Burgos, Spain, MIS 9) were analysed. The data show that some butchery activities exhibit variability as a result of multiple conditioning factors and, therefore, the identification of cultural patterns through the resulting cut-marks presents additional difficulties. However, other activities, such as marrow removal by means of intentional breakage, seem to reflect standardised actions unrelated to the physical characteristics of the bones. The statistical tests we applied show no correlation between the less dense areas of the bones and the location of impacts. Comparison of our experimental series with the archaeological samples indicates a counter-intuitive selection of the preferred locus of impact, especially marked in the case of Bolomor IV. This fact supports the view that bone breakage was executed counter-intuitively and repetitively on specific sections because it may have been part of an acquired behavioural repertoire. These reiterations differ between levels and sites, suggesting the possible existence of cultural identities or behavioural predispositions dependant on groups. On this basis, the study of patterns could significantly contribute to the identification of occupational strategies and organisation of the hominids in a territory. In this study, we use faunal data in identifying the mechanics of intergenerational information transmission within Middle Pleistocene human communities and provide new ideas for the investigation of occupational dynamics from a zooarchaeological approach.

## Introduction

Archaeological records are composed of multiple individual actions, which lead to the creation of variability in the assemblages, either as idiosyncratic features or traits submerged in repetitive patterns [1]. From this perspective, Binford [2] provides a scientific frame of reference, championing the system and the group as units of analysis. Therefore, the processing of animal resources and storage decisions are analysed as outcomes of group behaviour [3], [4].

Social learning and imitation are information-gathering mechanisms based on the experience or behaviour of other individuals. They allow the existence of an intergenerational transmission of social type and a stabilisation of cultural forms, which, in turn, strengthens the behavioural traditions of human groups [5]–[12]. Imitation can be considered as an alternative social learning mechanism, which reflects the characteristics of a specific demand for different types of cultural products and enables a highly reliable transmission of the information [13], [14]. During imitation, active communication between the user and the observational learner is not always necessary, as the reproduction of an event can be based on passive observation. Observation leads to the reproduction of an action, the perpetrator of which does not have to understand its aim *a priori*; this is known as cognitive “opacity”. Understanding the process and its aim is a subsequent result, which culminates in an assimilation of relevant concepts, causes and consequences that are accepted and eventually learned by the observer. Cognitive “opacity”, implicit in imitation contexts, slows the process of comprehension and represents a structural learning ability problem in early forms of socio-cognitive transmission mechanics, thereby impeding the cultural reproducibility of new practices that were reliant on it. For Gergely and Csibra [13], this circumstance meant an evolutionary pressure on early hominids culminating in the selection of a new type of socio-cognitive learning mechanism that would ensure the fast and efficient transmission of information. This new system, human pedagogy, favoured a new mechanism, so that learning via imitation would be guided [15], [16]. Within this process, communication through language could favour the transfer of culturally relevant knowledge in a highly effective way. The use of pedagogy implies the existence of cognitive resources on the part of both participants in the communicative process, which guarantees the selective efficiency of the cultural knowledge.

The anatomical adaptations related to the presence of a highly efficient oral communication system (anatomical structure of the basicranium, morphological and metric variation in the hyoid bone and auditory capacities) seem to be present in the human fossil material from Middle Pleistocene sites in Africa and Europe, as well as from Neanderthal specimens [17]–[19]. In this way, the hominids of this period would have had the capacities for repeating actions, assimilating concepts with a particular objective and for learning from other members of the group who “knew” certain processes or activities. Consequently, it is reasonable to hypothesise that within a group certain traditions would culminate at the archaeological level in standardised patterns. These patterns may be different from those developed by other groups and could foster the existence of certain group or territorial entities. According to Enloe [20], present-day hunter-gatherer subsistence varies in organisational strategy, which should be manifested culturally in the patterning of faunal remains. From ethnographical observations, Yellen [21] states that there are culturally prescribed rules, which have no cross-cultural type of logic behind them and which might be expected to vary from group to group. To evaluate this idea, we have studied several faunal assemblages from Bolomor Cave (Valencia, Spain) and a sample from the TD10-1 sublevel of Gran Dolina (Sierra de Atapuerca, Burgos, Spain). Some of these sets show well standardised long bone breakage patterns, which express the concept of the individual as a knowledgeable actor, able to influence outcomes through involvement in a social context. Our endeavour, therefore, is to use faunal data to place the individual as a member of a group in the landscape and at their evolutionary stage, showing how bones carry more information than just dietary knowledge. This fact could open up another perspective on the Middle Pleistocene faunal data, which all too often are regarded as too meagre to answer social questions, and help to interpret occupational dynamics during the formation of the archaeological sites.

## Methodology

In order to study the faunal assemblages from Gran Dolina TD10-1 and Bolomor Cave, we have followed a methodological approach developed in the zooarchaeology discipline, with a special focus on anthropogenic damage produced during the nutritional phase of carcass use. To assess completeness of the sample, NR (Number of remains) or NISP (Number of Identified Specimens), MNE (Minimum Number of Elements), MNI (Minimum Number of Individuals) and MAU (Minimal Anatomic Units) with their respective percentages have been calculated [22], [23]. Both Gran Dolina TD10-1 and Bolomor Cave contain a significant volume of remains that are not taxonomically identifiable. To include the remains with the identified specimens, weight categories were established following the criteria introduced by Bunn [24], which were translated into the metric system and grouped into five animal body size classes.

Surface alterations generated by the hominids are treated at both macroscopic and microscopic levels. For microscopic study, an Olympus Europe SZ11 (magnification up to 110) and Environmental Scanning Electron Microscope -ESEM- (FEI QUANTA 600) were used. The anthropogenic damage observed on the faunal remains includes cut-marks, intentional bone breakage, burning and human tooth-marks. In order to identify possible standardised processes on bone remains, the location and distribution of modifications in terms of anatomical area and region (portion and side) were registered. These bone landmarks are appropriately standardised for ease of consideration using a devised numerical system. This system divides the long bones into five portions, with portion 1 being the most proximal part of the skeletal element (proximal epiphysis) and portion 5 the most distal (distal epiphysis). Portions 2, 3 and 4 are located on the diaphysis, in which portion 2 is the most proximal part (proximal metadiaphysis), portion 3 is the medial region (mid-shaft), and portion 4 is the most distal part (distal metadiaphysis).

Cut-marks have been identified according to the criteria established by Binford [4], Potts and Shipman [25], Shipman and Rose [26], Bromage and Boyde [27], Shipman et al. [28] and Noe-Nygaard [29]. Three types of cut-marks have been identified and grouped into incisions, scrapes and chop-marks. These three types differ according to the manner in which human groups used stone tools [23], [26], [30]. The analysis of cut-marks took into account the number of striations, location on the anatomical element, distribution over the surface (isolated, clustered, crossed), orientation with respect to longitudinal axis of the bone (oblique, longitudinal, transverse) and delineation (straight or curved). Measurements of each striation (maximum and minimum length) were taken in millimeters using a digital calliper and in some cases, a stereoscopic microscope. The different types of cut-marks have been associated with specific butchering activities following the observations carried out by Binford [4], [31], Fisher [32], Domínguez-Rodrigo [33], [34], Nilssen [35] and Lyman [23].

Bone breakage is classified following the criteria established by Bunn [36] and modified by Villa and Mahieu [37]. The outline (transverse, curved/V-shaped, longitudinal), fracture angle (oblique, right, mixed) and surface edge (smooth, jagged) is recorded. Bone breakage on the small animal bones was analysed and classified as old (occurring at or near the time of deposition) or new (occurring during or after excavation) [38]. This last type was well defined by colour changes in the section of bone and the outline and fracture angle.

Surface damage caused during bone breakage was also analysed and the diagnostic elements of anthropic breakage were documented on faunal remains. These modifications included percussion pits or percussion marks, percussion notches or conchoidal scars, impact flakes, adhering flakes and peeling. Percussion notches are semicircular shaped indentations on fracture edges with corresponding negative flake scars [39], [40]. Impact flakes refer to positive flakes of the percussion notches and display the same basic technical attributes as stone flakes (mainly ventral face with point of detachment and bulb). Percussion pits or percussion marks are often closely associated with patches of striae that result from slippage of stone against bone during impact events [40], [41]. Peeling defines a roughened surface with parallel grooves or fibrous texture produced when fresh bone is fractured and peeled apart, similar to bending a small fresh twig with two hands [42]. These collected data were compared with published arguments regarding the revision of criteria for distinguishing between percussion and carnivore tooth-marks and geochemical etching and bioerosion [43]–[46]. In order to observe possible reiterations, descriptions include the location of the damage on the anatomical element (portion and side) and the distribution over the surface. To check the existence of a possible correlation between location of percussion impacts and bone mineral density, different bivariant tests (Spearman’s *rho* and Kendall’s *tau*) were employed, taking into account each skeletal element portion (epiphysis, proximal and distal metadiaphysis and mid-shaft). For calculation of this, bone density data estimated by Lyman [22] and Lam et al. [47] have been used. For discrete multivariate data, we have applied the implementation of the Exact Multinomial Test in EMT library of the statistical package R and the function “multinomial.test”. For testing differences of categorical groups with factors in contingency tables, we have used the Fisher’s exact test (FET) (see Text S1 for more details). These tests were graphically complemented with a correspondence analysis. An asymmetric graph was used with rows in the principal coordinates and columns providing the standardized residuals. The “ca” R library was selected for this purpose.

Burning damage is used here in terms of present/absent and based mainly on colour changes (mainly from brown, black, and grey to white). Bones exposed to fire exhibit modifications differentially, including colour changes, cracking, fractures and shrinkage (e.g.,[48]–[53]). The most obvious alterations are changes in the natural coloration of bone material. As in the case of cut-marks and percussion marks, the anatomical area and region of alteration by burning are also registered. We designate degrees of alteration by burning according to six categories of intensity, degree 0 being the unburned bones and degree 5 the calcined remains [54].

### Archaeological Approach: TD10-1 of Gran Dolina and Bolomor Cave

#### Gran dolina TD10-1

Gran Dolina is a large cavity located in the Sierra de Atapuerca (Burgos, Spain). Its stratigraphic succession of up to 18 m high was initially divided into 11 stratigraphic units called TD1 to TD11 from bottom to top, which was slightly revised in subsequent studies [55]–[57]. Palaeomagnetic data placed the Matuyama-Brunhes boundary at the top of level TD7, which divides the stratigraphic sequence into an Early Pleistocene section (TD1-2 to TD7) and a Middle Pleistocene section (TD8 to TD11). The TD10 level is the most recent deposit at the site with archaeo-paleontological remains. Its sediments are composed of sands with gravel and limestone clasts [55]. TD10 is divided into four litho-stratigraphic sub-units: TD10-1 (it includes TD10-sup), TD10-2, TD10-3 and TD10-4 in the base. A variety of dating methods (U/Th, ESR, TL, IRSL) have been applied at the site. Available geochronological studies in TD10 provided a TL date of 379±57 ky for the bottom of TD10-1, and a mean date of 337±29 for its top [57]–[60]. From a technological point of view, TD10-1 is classified as a transitional moment between Mode 2 or Acheulean and Mode 3 or Mousterian [60]–[62]. Flakes, denticulates and side-scrapers are the most common elements. Lithic refitting related to production sequences indicates mainly short and incomplete knapping activities at the site [63]. All the used raw materials (two types of chert, quartzite, quartz, sandstone and limestone) are found within a 5 km radius of the site [64]. Bone is occasionally exploited to make artefacts, both directly (bone hammer) and previously configured (side-scrapers) [65].

Regarding the faunal remains, the sample from TD10-1 analysed here (2000–2001 excavation season) is composed of 22 taxa, with *Cervus elaphus*, *Equus ferus* and *Oryctolagus* sp. as dominant species. Adult specimens determine the profile based on the age at death (MNI = 40 adults of 60 individuals) (Table 1). The proportion of long bone fragments (NR = 5728 of 11081 remains) is slightly higher than flat bones (NR = 4066/11081). Among the long bones, the assemblage consisted of mainly long bone shaft fragments (NR = 3950 of 5728 long bone fragments), which are not always identifiable taxonomically. From %MAU data, the skeletal representation of ungulates is characterised by the abundance of stylopodials (femur and humerus) (61.8), zeugopodials (radius and tibia) (51.8) and mandibles (62.5) and by a low representation of the axial skeleton (vertebrae and ribs) (8.5) [66]. The elements with the greatest marrow value are those with the greatest representation. This phenomenon could be interpreted as the product of anthropogenic selective transport [4], [67].

**Table 1:** NR, NISP, MNE and MNI by ages from the TD10-1 faunal assemblage.

Taxa	NR	NISP	MNE	MNI	neo	inf	juv	ad	sen
*Ursus arctos*	3	3	2	1				1	
*Canis lupus*	10	10	6	2		1		1	
*Vulpes vulpes*	16	16	13	2		1		1	
*Panthera leo fossilis*	17	17	15	1				1	
*Lynx* sp.	1	1	1	1				1	
*Hystrix* sp.	2	2	1	1				1	
*Stephanorhinus* cf. *hemitoechus*	52	52	9	2			1	1	
*Equus* ferus	260	260	62	9		2	3	3	1
*Equus* cf. *hydruntinus*	12	12	5	2			1	1	
*Sus scrofa*	1	1	1	1				1	
Cervidae indet.	121	121	24	2	1	1			
*Megaloceros giganteus* ?	1	1	1	1				1	
*Dama dama clactoniana*	2	2	2	1				1	
*Cervus elaphus*	762	762	232	9		1	1	6	1
*Bison* sp.	144	144	55	5		1	1	2	1
*Hemitragus bonali*	5	5	5	1				1	
*Capreolus capreolus*	3	3	3	2		1		1	
Erinaceidae	11	11	8	1				1	
*Oryctolagus* sp.	329	329	167	12		1		11	
Aves, unident.	7	7	2						
Passeriformes	25	25	18	1				1	
Phasianidae	9	9	8	1				1	
Corvidae	17	17	16	1				1	
Pisces	1	1	1	1				1	
Very large size	101		11						
Large size	1432		97						
Medium size	4726		202						
Small size	2342		159						
Very small size	32		13						
Unident.	637								
Total	11081	1811	1139	60	1	9	7	40	3

**Figure 1:**
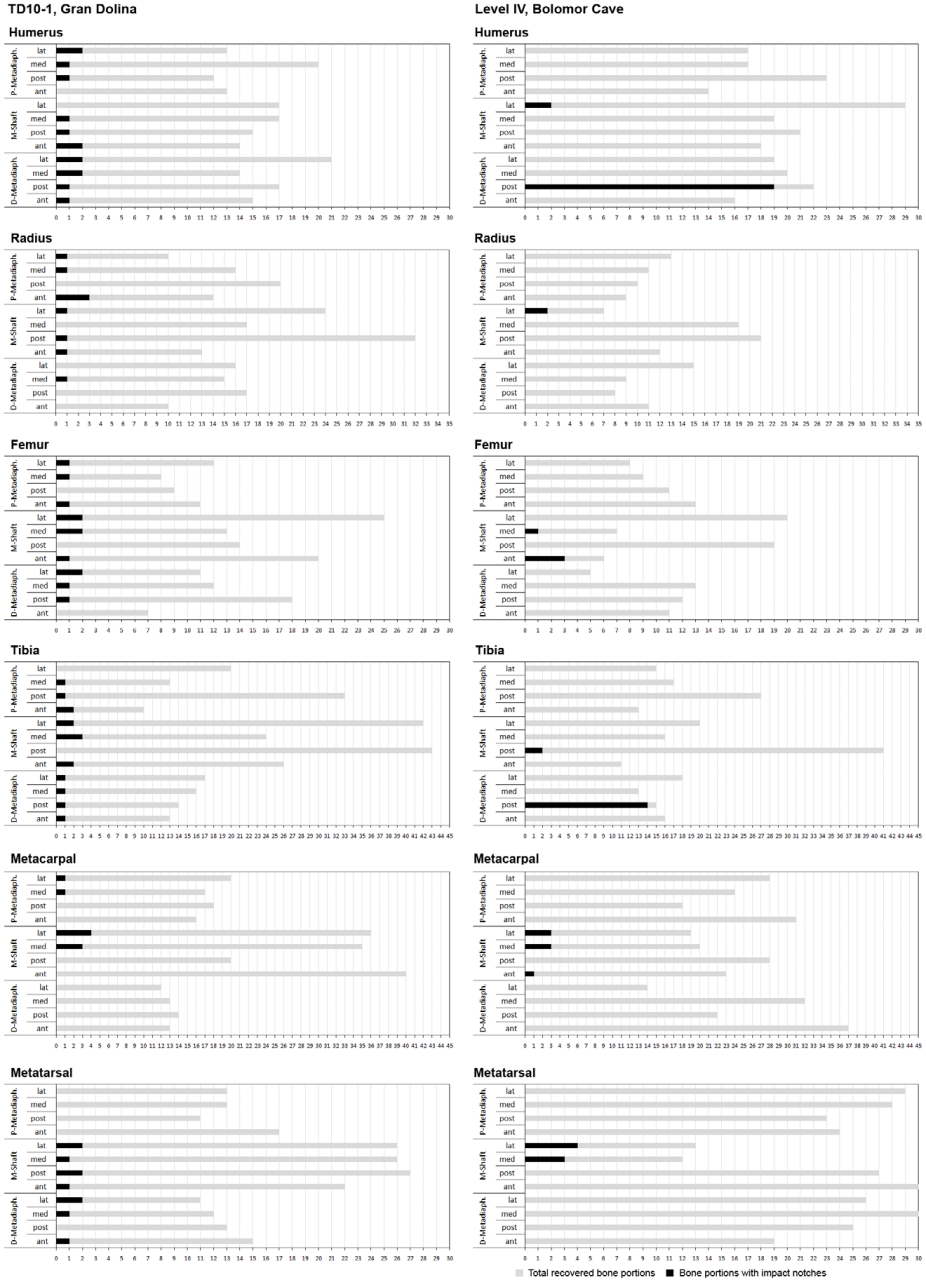
Graphical representation showing the relationship between the total of recovered bone portions and bone portions with impact notches for the TD10-1 faunal assemblage and level IV of Bolomor Cave by skeletal elements belonging to the appendicular skeleton of ungulates. P = Proximal; M = Mid; D = Distal; lat = lateral; med = medial; post = posterior; ant = anterior.

Analysis of bone breakage shows that the curved/V-shaped predominates, along with oblique angles and smooth edges [66]. The degree of bone breakage at TD10-1 is related to green bone breakage, according to the criteria established by Bunn [36] and Villa and Mahieu [37] (Table 2). Diagnostic elements of intentional anthropogenic breakage are recognised on 1329 bone fragments. These diagnostic elements are mainly percussion notches (121) and impact flakes (1166). Percussions notches on bone fragments identified anatomically show a high diversity in location (in terms of portion and overall surface) (Figure 1; Table 3). Actually, the assumption of uniformly distributed notches could not be discarded for any of the six bones under consideration after applying Exact Multinomial Tests to the observed distributions. Metacarpus was the worst-fitted case (p-value = 0.2045) (Table S1). In cases of small prey, fragmentation registers as transverse and curved-shaped fracture outlines close to the ends of limb bones and at oblique and mixed angles. For several researchers, this type of breakage is usually found in anthropogenic contexts and corresponds to green-bone fractures [68], [69], [70], [71], [72]. In addition, shaft cylinders (limb bone-shaft fragments with their full circumference) have been recovered (NISP = 35 of 113 belonging to stylopodials and zeugopodials) together with a significant proportion of extremities (NISP = 67/113).

**Table 2:** Frequencies of fracture outlines, fracture angles, fracture edges and shaft circumferences for long bone remains (≥2 cm) from the Gran Dolina and Bolomor Cave sites according to the criteria established by Villa and Mahieu [37].

		Gran Dolina	Bolomor			
		TD10-1	XVIIc	XVIIa	XI	IV
**No. Fractures**		10081	3391	1756	492	7758
**Fracture outline**	Transverse (%)	1619 (16.1)	659 (19.4)	365 (20.8)	114 (23.2)	1684 (21.7)
	Curved/V-shaped (%)	5608 (55.6)	1606 (47.4)	822 (46.8)	253 (51.4)	3681 (47.4)
	Longitudinal (%)	2854 (28.3)	1126 (33.2)	569 (32.4)	125 (25.4)	2393 (30.8)
**Fracture angle**	Oblique (%)	5548 (55)	1557 (45.9)	779 (44.4)	235 (47.8)	1934 (24.9)
	Right (%)	1985 (19.7)	1062 (31.3)	563 (32.1)	194 (39.4)	1932 (24.9)
	Mixed (%)	2548 (25.3)	772 (22.8)	414 (23.6)	63 (12.8)	3892 (50.2)
**Fracture edge**	Smoothed (%)	9079 (90.1)	2910 (85.8)	1501 (85.5)	422 (85.8)	5835 (75.2)
	Jagged (%)	1002 (9.9)	481 (14.2)	255 (14.5)	70 (14.2)	1923 (24.8)
**Shaft circumference**	≤1/4	2364	299	378	81	1854
	1/4–1/2	409	97	94	37	360
	1/2–3/4	85	8	7	7	22
	≥3/4	9				

**Table 3:** Notch distribution patterns on the main long bone (humerus, radius, femur, tibia and metapodials) belonging to ungulates from the Gran Dolina TD10-1 and Bolomor Cave faunal assemblages.

		Gran Dolina			Bolomor											
		TD10-1			XVIIc			XVIIa			XI			IV		
		NR	Portion	Side	NR	Portion	Side	NR	Portion	Side	NR	Portion	Side	NR	Portion	Side
Humerus	*S.*cf. *hemitoechus*	1	2	lat												
	*Equus ferus*	2	4	med, lat										1	4	post
	*M. giganteus*				1	4(3)	lat	1	3	lat						
	*Dama* sp.				1	4	lat							2	4	post
	*Cervus elaphus*	10	2,3,4	ant,post,med,lat	4	4	lat	3	4	med				13	3,4	lat,post
	*Bison* sp.	1	4	lat												
	*Bos primigenius*													2	4	post
	*H. cedrensis*													1	4	post
	Very large size													1	3	lat
	Large size													1	4	post
Radius	*Cervus elaphus*	6	2,3,4	post,med,lat	2	3	ant	1	3	med	1	3	lat	1	3	lat
	*Bison* sp.	3	2,3	post,ant,med												
	*Bos primigenius*													1	3	lat
	Medium size							1	3	ant	1	4	ant			
Femur	*Equus ferus*	1	3	lat				1	3	ant						
	*Cervus elaphus*	8	2,3,4	ant,post,med,lat	2	3	med	1	3	ant	1	3	med	2	3	ant,med
	*Cervidae* unident.	1	4	lat												
	*Bison* sp.	1	3	med												
	*Bos primigenius*													1	3	ant
	Small size	1	3	lat										1	3	ant
Tibia	*Equus ferus*	1	2	med				2	3	post,med				4	3,4	post
	*Cervus elaphus*	12	2,3,4	ant,post,med,lat	3	3	med	3	3	lat	1	3	med	9	4	post
	*Bison* sp.	1	2	post												
	*Bos primigenius*													2	4	post
	*H. cedrensis*													1	3	post
	Small size	1	3	lat												
Mtc	*Equus ferus*													1	3	med
	*Cervus elaphus*	9	2,3	lat,med	2	3	lat	1	3	lat				3	3	lat
	*Bos primigenius*													3	3	ant,med
	*H. bonali*										1	3	lat			
Mtt	*Equus ferus*	1	3	lat				1	3	lat				1	3	med/lat
	*M.giganteus*										1	3	lat/med			
	*Dama* sp.							1	3	lat						
	*Cervus elaphus*	9	3,4	ant,post,med,lat				2	3	lat,post				5	3	lat,med
	*Bos primigenius*													1	3	med
	*H.bonali*				1	3	lat									

Mtc = Metacarpal; Mtt = Metatarsal; ant = anterior or cranial; post = posterior or caudal; med = medial; lat = lateral. Each long bone was divided into five different portions: proximal epiphysis (1), proximal metadiaphysis (2), mid-shaft (3), distal metadiaphysis (4) and distal epiphysis (5).

Cut-marks are documented on 584 bone fragments. These are mainly located on the remains of large and medium-sized animals. Although incisions show variability, it is possible to distinguish two groups based on their type and location (Table S2). Oblique and longitudinal incisions are mainly situated on limb bone diaphyses, and transversal sawing marks on areas that present difficulties for the extraction of soft tissues, such as attachment areas for muscles or tendons, insertions, crests or tubercles. Short and deep striations, related to dismemberment and disarticulation of the anatomical portions, are also identified on some epiphyses. With regard to skinning, animals are often skinned from the skull to the metapodials and in some cases up to the second phalanx. Scraping marks are often related to periosteum removal, although they may also arise from the excision of meat remnants from bones during surface preparations for subsequent breakage events. From the EMT, the p-values (<0.05, so the results differ significantly from the *ab-initio* model) indicate patterning for the humerus, femur and tibia, with cut-marks clustered preferentially on mid-shafts (Text S2).

Several carnivore remains are also recovered in the analysed sample from TD10-1: *Ursus arctos*, *Canis lupus*, *Vulpes vulpes*, *Panthera leo fossilis* and *Lynx sp.* Some of these predators were processed by human groups (a lion and a fox) while others may have been introduced naturally into the cave, or even been brought in by other carnivores. The overall record of tooth-marks on bones supports the view that predatory animals frequented the cave in order to scavenge remains abandoned by human groups and probably sought refuge or made dens between intervals of human occupation [66], [73], [74].

#### Bolomor cave

Bolomor Cave is located on the southern slope of the Valldigna valley (Valencia, Spain). The site is comprised of an elevated rock-shelter approximately 100 m above sea level. The stratigraphic sequence is divided into 17 levels, with numbering commencing from the top of the deposit, and with a maximum thickness of 14 m [75]. The karstic deposition has been dated by AAR and TL to between MIS 9 and MIS 5e [76].

The lithic industry from Bolomor Cave is classified as a Middle Palaeolithic techno-complex. This techno-complex is older than the regional Classic Mousterian age and has its beginning at some point during the Middle Pleistocene, under the consideration of an Ancient Middle Palaeolithic, although it is not related to the Acheulian period [77]. Used raw materials mainly consist of flint and, to a lesser extent, of limestone and quartzite. As a distinctive feature, the level IV industry mainly includes small tools, predominantly scrapers, denticulates and various retouched pieces, which are characterised by intensive re-use and recycling [78].

Several combustion structures have been documented at levels II, IV, XI and XIII. The hearths are morphologically simple and are not superimposed, they have a lenticular appearance with diameters between 30–120 cm and an average thickness of 5–10 cm [79].

The Bolomor faunal record includes more than 30 species belonging to the categories of Cercopithecinae, Carnivora, Ungulata and small prey (Leporidae, Aves, Testudinidae, Amphibia and Salmonidae). *Cervus elaphus* and *Oryctolagus cuniculus* are the most represented taxa, along with *Aythya* sp. at level XI and *Testudo hermanni* at level IV. In the age at death profile for the individuals recovered (MNI), adults clearly predominate (XVIIc = 23 adults of 30 individuals; XVIIa = 35/38; XI = 24/30; IV = 83/99) (Table 4, Table 5). The proportion of long bone fragments (NR) (XVIIc = 550 of 1307 remains; XVIIa = 677/1732; XI = 409/1047; IV = 16657/25323) is higher than flat bones (XVIIc = 214 of 1307 remains; XVIIa = 317/1732; XI = 231/1047; IV = 6286/25323). The Bolomor assemblages consisted of mainly long bone shaft fragments (XVIIc = 369 of 550 long bone fragments; XVIIa = 406/677; XI = 325/409; IV = 14984/16657). According to %MAU, the assemblages are mainly composed of cranial elements (mandibles and maxillaries) (XVIIc = 56.1; XVIIa = 51.6; XI = 51.8; IV = 71.8) and proximal appendicular bones (XVIIc = 60.4; XVIIa = 60.5; XI = 39.5; IV = 75.9) in ungulates. The axial skeleton is underrepresented in all levels (XVIIc = 1.8; XVIIa = 4.8; XI = 2.2; IV = 6). For small prey, such as lagomorphs, all the skeletal elements are represented, although hind and fore limbs predominate over all other skeletal parts at level IV (XVIIc = 44.8; XVIIa = 64.2; XI = 57.1; IV = 40.6), in addition to scapular and pelvic girdles at level XI and pelvic girdles at level XVII (scapula: XI = 57.1/pelvis: XI = 42.9; XVIIc = 47.2; XVIIa = 73.3). Regarding birds, the anatomical elements with the highest survival are coracoid and tibiotarsus at level XI and IV (coracoids: XI = 93.7; IV = 63.3/tibiotarsus: XI = 87.5; IV = 46.7). In the case of tortoises, the anatomical elements most abundant are humeri (IV = 94.7), femuri (XI = 50; IV = 73.7) and plastron (XI = 100; IV = 68.4) [66].

**Table 4:** NR, NISP, MNE and MNI by ages from the Bolomor faunal assemblages: levels IV and XI.

	IV							XI					
Taxa*	NR	NISP	MNE	MNI	imm	ad	sen	NR	NISP	MNE	MNI	imm	ad
*Macaca sylvana*	1	1	1	1		1							
Carnivora unident.	5	5	4										
*Ursus arctos*	1	1	1	1		1							
*Canis* cf. *lupus*	2	2	2	1		1							
*Vulpes vulpes*	2	2	2	1		1							
*Panthera leo*	3	3	2	2	1	1							
*Lynx pardina*	2	2	2	1		1							
*Castor fiber*								2	2	1	1		1
*Palaeoloxodon antiquus*	4	4	2	1	1								
*Stephanorhinus hemitoechus*								3	3	2	2	1	1
*Equus ferus*	65	65	25	4	1	3		2	2	2	1		1
*Equus hydruntinus*	16	16	9	1		1							
*Hippopotamus amphibius*	46	46	5	2	1	1							
*Sus scrofa*	115	115	55	5	2	2	1						
*Megaloceros giganteus*								2	2	2	1		1
*Dama* sp.	91	91	41	3	1	2		4	4	4	1		1
*Cervus elaphus*	647	647	193	12	2	10		55	55	35	4	2	2
*Bos primigenius*	213	213	63	4	1	3		2	2	2	1		1
*Hemitragus bonali*								16	16	13	2	1	1
*Hemitragus cedrensis*	121	121	47	3	1	2							
*Oryctolagus cuniculus*	789	789	440	20	4	16		262	262	150	7	2	5
Passeriforme	25	25	21	2		2							
Galliformes	19	19	16	1		1							
Phasianidae	24	24	16	2		2							
*Anas* sp.	29	29	25	2		2							
*Aythya* sp.	34	34	28	3		3		202	202	167	8		8
Corvidae	20	20	13	1		1							
*Pyrrhocorax* sp.	6	6	6	1		1							
*Columba* sp.	34	34	25	2		2							
Strigidae	1	1	1	1		1							
Aves, unident.	17	17	2										
*Testudo hermanni*	526	526	131	19		19		4	4	3	1		1
*Bufo* sp.	4	4	2	2		2							
Pisces	2	2	2	1		1		1	1	1	1		1
Very large size	37		6										
Large size	1975		49					16		4			
Medium size	10274		116					128		14			
Small size	9053		275					247		20			
Very small size	304		61					92		8			
Unident.	816							9					
Total	25323	2864	1689	99	15	83	1	1047	555	428	30	6	24

* Human remains have not been included in this study.

**Table 5:** NR, NISP, MNE and MNI by ages from the Bolomor faunal assemblages: sublevels XVIIa and XVIIc.

	XVIIa						XVIIc					
Taxa	NR	NISP	MNE	MNI	imm	ad	NR	NISP	MNE	MNI	imm	ad
*Canis* cf. *lupus*	4	4	4	1		1						
*Palaeoloxodon antiquus*	2	2	1	1		1	2	2	1	1	1	
*Stephanorhinus hemitoechus*	8	8	3	2	1	1	1	1	1	1		1
*Equus ferus*	77	77	30	2		2	56	56	22	1		1
*Megaloceros giganteus*	10	10	9	1		1	8	8	5	1		1
*Dama* sp.	27	27	20	1		1	13	13	10	1		1
*Cervus elaphus*	177	177	58	4	1	3	132	132	47	4	1	3
*Bos primigenius*	24	24	13	1		1	22	22	13	1		1
*Hemitragus bonali*	28	28	20	1		1	6	6	6	2		2
*Oryctolagus cuniculus*	620	620	346	15	1	14	457	457	234	12	5	7
*Lepus* sp.	3	3	3	1		1						
Passeriforme	5	5	5	1		1	9	9	9	2		2
Galliformes	8	8	7	2		2						
Phasianidae	18	18	14	3		3	10	10	9	2		2
Anatidae	4	4	4	1		1						
*Anas* sp.							16	16	14	2		2
*Bufo* sp.	1	1	1	1		1						
Very large size							8		1			
Large size	186		11				219		11			
Medium size	364		22				235		16			
Small size	160		24				95		11			
Very small size							8		1			
Unident.	6						10					
Total	1732	1016	595	38	3	35	1307	732	411	30	7	23

**Figure 2:**
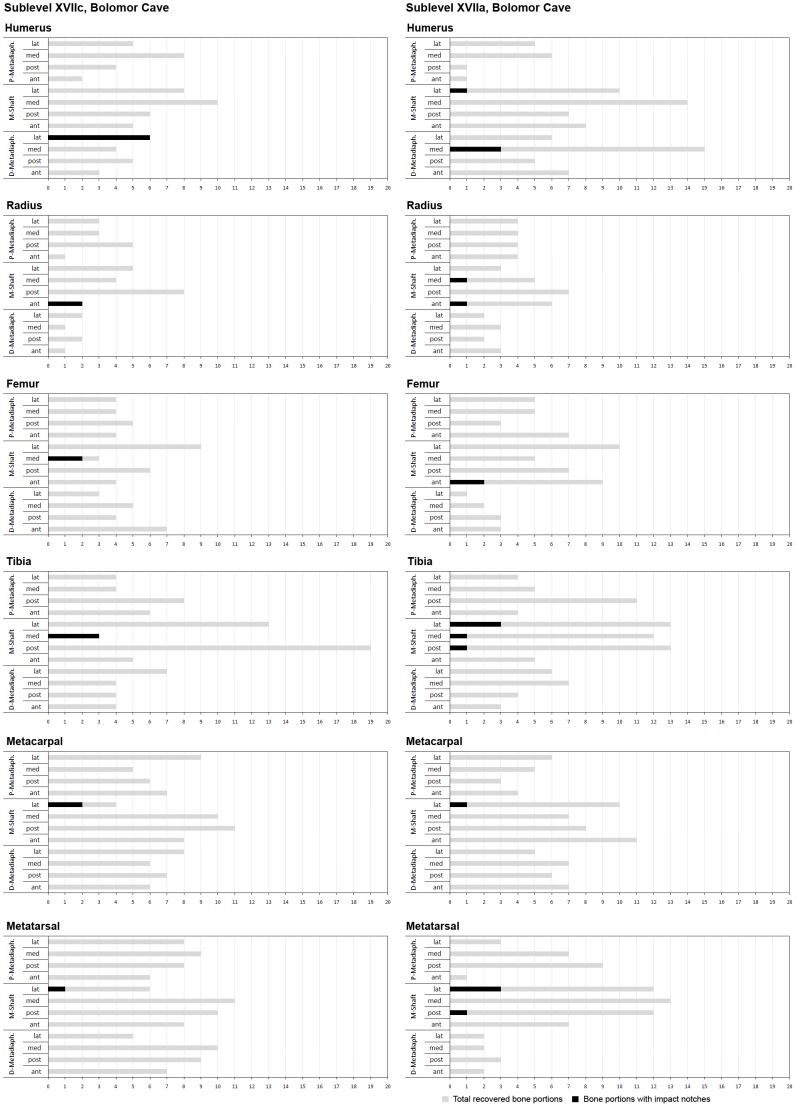
Graphical representation comparing the relationship between the total of recovered bone portions and bone portions with impact notches for the XVIIc and XVIIa faunal assemblages from Bolomor Cave by skeletal elements belonging to appendicular skeleton of ungulates. P = Proximal; M = Mid; D = Distal; lat = lateral; med = medial; post = posterior; ant = anterior.

Bone breakage indicates that curved/V-shaped fractures, oblique angles and smooth edges are predominant in the Bolomor faunal record (Table 2). According to Bunn [36] and Villa and Mahieu [37], these patterns indicate that the bone was mainly fresh when broken. Evidence for anthropogenic bone breakage is documented on 82 ungulate remains at sublevel XVIIc, 117 at sublevel XVIIa, 57 at level XI and 839 at level IV. The most abundant diagnostic elements are percussion notches (XVIIc = 31; XVIIa = 28; XI = 6; IV = 112) and impact flakes (XVIIc = 48; XVIIa = 79; XI = 47; IV = 676). Percussions notches on the identified skeletal elements show a high standardisation in location (in terms of portion and side) depending on the archaeological level (Figure 1; Figure 2; Table 3). This phenomenon is especially pronounced at level IV, which presents the highest number of remains with diagnostic elements of anthropogenic breakage and shows distributions statistically different from a uniformly distributed case (i.e., when Exact Multinomial Tests were applied to each bone, we obtained a p-value <0.05 for the radius and p-values <0.001 for the rest of cases) (Table S1). For instance, the ungulate humeri (both Artiodactyla and Perissodactyla) from level IV contain percussion notches on the posterior side of the distal metadiaphysis in 19 bone fragments of the 21 recovered. Similarly, the ungulate tibiae show impact points on the posterior side of the distal metadiaphysis in 14 of the 16 registered remains.

A different case is observed on small prey. No diagnostic elements of fracturing by active or passive percussion are identified on their remains. Nevertheless, the fragmentation is present both on the fore and hind limb in the form of transverse and curved/V-shaped fracture outlines close to the ends and oblique and mixed angles. The breakage near the ends is not common in non-anthropogenic or post-burial contexts [68]–[72]. Following the study carried out by Cochard [69], Cochard et al. [70] and Sanchis Serra [80], the curved/V-shaped fractures are features of breakage on fresh avian and leporid bones. In order to fracture the small prey bones, the human groups of Bolomor probably combined the actions of their hands and teeth. As a result of such combinatory actions, well-established patterns can be observed on different skeletal elements in the form of shaft cylinders (NISP XVIIc = 31 of 132 belonging to stylopodials and zeugopodials; XI = 15/138; IV = 71/182) and isolated ends (NISP XVIIc = 95/132; XI = 50/138; IV = 51/182). Following the criteria described by Cochard [69], Laroulandie [71], Pérez Ripoll [72], Sanchis Serra [80], Landt [81] and Lloveras et al. [82], human tooth-marks can be identified on rabbit, bird and tortoise remains at Bolomor. These marks are associated with fracture edges and, in some cases, they form crenulated edges or peeling [66], [83]–[86].

Cut-marks are documented on 119 faunal remains at sublevel XVIIc, 117 at sublevel XVIIa, 79 at level XI and 1817 at level IV. Striations are mainly identified on the long bones of medium and large-sized animals, with the majority of the incisions on the diaphysis, and the sawing marks on the metaphysis (Table S3). The EMT p-values show no indication of patterning for most long bones from Bolomor XVIIc, XVIIa and IX (p-value >0.05), in contrast with the cut-mark distribution on long bones from Bolomor IV (p-value = 0.000) (Text S2). Whereas this may be suggestive of random placement of cut-marks on the Bolomor levels (other than Bolomor IV) it actually has more to do with sample size. The number of cut-marks reported for Bolomor XVIIc,a and IX is substantially smaller than those reported for Bolomor IV. Given that the EMT tests are very conservative, it is not surprising to detect no pattern when the sample size is small. In contrast, the larger sample of cut-marks for Bolomor IV indicates that patterning exists, since cut-marks are cluster mainly on mid-shafts instead of on metadiaphyseal sections.

Burning is identified on 645 faunal remains at level XI and 15585 at level IV. This modification is observed on every type of skeletal element, with a definite predominance of long bones of medium and small-sized animals at level IV and of vertebrae and tibiae at level XI. In the case of lagomorphs and birds, the highest grades of burning on bones with double colouration coincide with the areas of the skeleton with less muscle (mainly joints of limb bones). In tortoises, the carapace is the most affected element, and degree 2 is the most abundant modification type. On the contrary, degrees 4 and 5 are practically non-existent in the assemblage. Double colouration on different sides is observed mainly on tortoise bones and, specifically, on the shell. The carapaces show a greater degree of burning on the dorsal surface than on the ventral surface in 84.61% of the cases [83].

The presence of carnivores at Bolomor Cave is rare. Nevertheless, fossil remains of *Ursus arctos*, *Ursus tibetanus*, *Canis lupus, Panthera leo, Lynx pardina*, *Vulpes vulpes* and *Meles meles* have been recovered. The tooth pit sizes on bone fragments according to bone type (cancellous and dense cortical) might be related mainly to small and medium-sized carnivores. The presence of non-human predators and their activities may suggest, with some exceptions (evidence for human processing on fox, lynx and lion at level IV), sporadic events of non-human predators that act as scavengers of hominid refuse [66], [85], [86].

## Discussion

### Patterns in the Faunal Processing Sequence

Following the time sequence that organises faunal processing into a hierarchy, four main actions can be distinguished: procurement, method of transporting the carcass, the processing and consumption techniques and the subsequent disposal of the remains. The different segments of the sequence are highly interrelated, and therefore the first step (the method and type of acquisition) greatly determines the subsequent exploitation sequence, thereby influencing the presence of marks on the bones. Taking into account that this circumstance may introduce variations or elements of distortion when standardised action sequences are being analysed in the faunal record, the procurement method should be tackled as a general dynamic within the assemblage using different elements. In the archaeological cases presented here, 1) the systematic proportion of skeletal elements with a high nutritional value, 2) the predominance of adult animals, 3) cut-marks related to the removal of the viscera and 4) oblique and longitudinal incisions on the diaphysis of the limb bones -which are associated with defleshing of large muscle masses- suggest that the access hominids had to the animals was mainly primary and immediate (see extensive discussion in [66]). These practices, nevertheless, might coexist with sporadic events of secondary access to the carcasses in the case of the TD10-1 sublevel and level IV [66], [86].

On this basis, Roebroeks [87] states the view that large-mammal hunting in the Middle Pleistocene of Europe must have been a co-operative activity only made possible by verbal communication and social interaction between individuals. In his opinion, such co-operation would have involved the exchange of information, most likely including language, between older and younger individuals. Thus, if it is possible to establish a standardised behaviour as a result of the existence of communication and learning for some techniques of hunting, why can this principle not be extended to the subsequent processing sequence?

The extraction of both the external (skin, tendons and meat) and internal (fat and marrow) resources of the bones could reflect the existence of standardisation during the processing of the carcasses. In that case, we could observe at the archaeological level what Yellen [21] terms “style” in the butchery and consumption of mammals in the !Kung Bushmen, or what Bunn calls “illogical ways” (in [88], [24]). For Yellen [21], the analysis of faunal remains can be used as a valuable tool to approach archaeological questions related to cultural relationships through time and space, i.e., “traditions”.

Cut-marks show the anthropogenic extraction of the external soft tissues and allow us to deduce the preparation of the carcass for subsequent consumption. Several studies have sought to identify butchery patterns among modern hunter-gatherer groups in order to explain behavioural questions (e.g, [89]–[93]). There remains, however, a long-standing debate on these matters, especially when results of the discussion are also invoked for interpretations of the early Pleistocene [94], [95]. Some authors use the same data on butchery to infer differentiated conclusions about anthropic access to the carcasses, which range from passive scavenging to primary access, passing through mixed strategies of early, intermediate and late access [96]–[102]. Some researchers question cut-mark frequency and distribution as a good way to establish the pattern of activity on carcasses [100] and others argue that opposite butchery behaviours may generate similar cut-mark patterns [103]–[105]. Additionally, this interpretative difficulty may be also be increased by the nature of faunal assemblages; i.e. the anthropogenic accumulations from Pleistocene archaeological sites are often the product of overlapped activities and/or occupations, which can involve disruptive processes, such as cleaning, transport or trampling. The results are palimpsests composed of multiple singular events that could make the archaeological interpretations difficult [106]. From this perspective, animals that compose the assemblages could reflect more than one method of acquisition and therefore, more than one technique of processing.

Cut-marks must be understood as accidents produced during the extraction of the external resources [93]. These accidents are generated when the cutting edge of the lithic tool comes into contact with the bone surface. Padilla [107] shows by means of butchery experiments that even highly skilled butchers intentionally deflesh carcasses with the aim of minimizing the number of cut-marks on bones leaving diagnostic traces in specific anatomical areas. Therefore, this evidence is not intentional and is subject to different conditioning factors, which appear to have a significant effect on the defleshing and disarticulation activities. We must take into account that the animals’ physical, morphological and physiological characteristics could condition the presence and reiteration of cut-marks on certain areas of bone. In this way, the hominids may focus more on areas where there are muscular insertions and tendons, or where bone morphology prevents the easy extraction of meat. Such factors could give rise to unintentional patterns on the bones during the defleshing process that seem to be guided not by an individual intention but, as Binford [3] argued, by animal anatomy. In addition, other multiple factors may influence butchery processes, such as the experience of the butcher, prey size, site functionality, seasonality, ground characteristics and/or available human technology -including boiling and metal tools- [89], [90], [92], [100], [108]–[110]. These variables affect behaviours and, in consequence, may produce a high variability in the resultant cut-marks. In spite of this, a patterning related to location of cut-marks is detected from the exact multinomial tests in those archaeological assemblages with larger samples -Gran Dolina TD10-1 and Bolomor IV- (Text S2). The reason for this is that, despite the bias in preservation of various sections (properly taken into account in the *ab-initio* model), cut-marks cluster preferably on mid-shafts instead of on metadiaphyseal sections. This pattern may be conditioned by the main type of access to the carcasses identified at both TD10-1 and Bolomor IV (primary and early). For Domínguez-Rodrigo and Pickering [101], these kinds of cut-marks could have resulted from hominids butchering fully fleshed carcasses, being inconsistent with other procurement modalities, such as passive scavenging. Nevertheless, the Fisheŕs exact test (FET) used to compare the cut-mark distribution in TD10-1 and in Bolomor IV shows significant differences between them (e.g., p-value = 0.040 for the tibia and p-value = 0.020 for the humerus). This phenomenon may be related to uncontrolled conditioning elements or variables involved in a complex interplay of cultural and non-cultural factors. With these ideas, we do not rule out the existence of a possible transmission of information for the development of defleshing, disarticulation or skinning, rather we note the difficulty involved in its archaeological identification. The movements made during these activities could be standardised as a result of learning; however, the cut-marks could be only registered on those areas that, due to above-mentioned conditioning factors, facilitate the contact of the tool with the bone. An example of this phenomenon is provided by the Nunamiut “butchery school”, where the pupil imitates as precisely as possible the master butcher in order to learn how to disarticulate the foot of a caribou [3]. During this process, knowledge is transferred as part of the relationship between master and pupil and overall, as a set of body techniques and movements, which are not always registered on bones. For Lyman [93], understanding the variability in cut-mark frequencies presents a great deal of difficulty if multivariate interpretative models are not tackled. According to this author, multiple and diverse elements can comprise the processing sequence, including fortuitous variables.

Burning alterations identified on the faunal remains may suggest the presence of several processes, such as thermal treatment of the meat, the preparation of bones to facilitate their breakage or the development of possible cleaning activities. Double colouration on the same bone surface has been recorded at levels XI and IV of Bolomor. The presence of these alterations suggests a differential preservation of the meat at the moment it is exposed to fire. The least affected areas are those that present a greater quantity of muscle and a lower degree of cremation, while the most affected are those that hardly have any tissue attached and therefore reach the highest degrees of colouration. This phenomenon allows us to infer that the meat was roasted prior to the removal of the bone. Despite this, double colouration on different sides is also documented. Their presence allows us to infer the existence of other processes, which may not be related to the cooking of the meat, such as cleaning activities or simply unintended actions that lead to the burning of the bones once they have been broken. This situation could hide the existence of patterns in a set and alter their possible standardisation. In spite of this, at times, it is possible to establish systematisation in roasting based on different degrees of coloration according to the sides. This is the case for shells of *Testudo hermanni* recovered at level IV of Bolomor [83]. Although there is a certain degree of variability, these alterations seem to describe a pattern based on the differential burning of the bone surface (with a greater degree of burning on the dorsal surface than on the ventral surface). This phenomenon has been interpreted as a result of cooking these animals before consumption. The characteristics of double colouration indicate that the tortoises could have been placed into the fire upside down. This pattern has been described ethnographically by Werner [111] for the preparation of tortoises among the Kayapó of Central Brazil. For several authors, these modifications represent elements that are diagnostic for the human consumption of tortoises [112]–[115].

After consideration of the different actions related to processing and consumption of external resources, bone breakage is perhaps the activity that best allows us to assess the presence of patterns in a Middle Pleistocene assemblage. The development of this process at the habitat places and the relative abundance of bone remains with elements that are diagnostic for this activity allow us to tackle this issue with guarantees. The ethnographic observations of Yellen [21], [88], [116] confirm that random is not the rule and that each limb bone is treated following a standardised process. However, and despite that the use of actualistic analogies can play a significant role in any attempt to understand archaeological evidence, comparative analyses between communities of modern hunter-gatherers and Pleistocene assemblages must be carried out with caution, given the evident differences related to technology and/or social systems. Gifford-Gonzalez [108] notices that the recent incorporation of cook-pots and boiling technology in some groups of current hunter-gatherers may substantially change the bone breakage patterns, which seem to adapt to items such as the size of container. Nevertheless, some activities related to the extraction of internal resources, such as the release of bone grease contained within spongy tissues, may be considered [117]. According to Outram [118], the systematic exploitation of marrow and grease by hitting, crushing or grinding, generates assemblages characterized by an almost total absence of elements with cancellous tissue (mainly epiphyses and vertebrae) and the presence of some diaphyseal cylinders, which could be turned into splinters by subsequent taphonomical processes. In our case study assemblages, appendicular epiphyses (NR TD10-1 = 592 whole and/or fragmented epiphyses of 5728 long bones; XVIIc = 62/550; XVIIa = 97/677; XI = 31/409; IV = 247/16657) and vertebrae (NR TD10-1 = 381; XVIIc = 26; XVIIa = 41; XI = 24; IV = 198) are recovered. In spite of their presence, axial elements show an underrepresentation in some animal body size classes (large and medium-sized ungulates at TD10-1 and large, medium and small-sized at Bolomor). This phenomenon has been interpreted as the product of human selective transport [4], [66], [67], [86]. But, to assess correctly the presence of epiphyses and axial bones in the anthropogenic Pleistocene assemblages, we must take into account the existence of post-depositional processes, the use of bone as fuel and, especially, the activities generated by carnivores during the secondary accesses. The smell of human refuse can attract many predators, which seem to act as scavengers in search of potentially consumable resources [4]. Several observations and experimental reproductions focused on this matter document a predilection for epiphyses of limb bones and elements of the axial skeleton (e.g., [119], [120]). The carnivore damage on these anatomical portions is so intense that in many cases they may make them disappear. In contrast, the diaphyses, which are generally highly fractured during the human processing and consumption, show little alteration. In the case of TD10-1 and Bolomor, carnivore damage is focused mainly on epiphyses, vertebrae and basipodials belonging to the most abundant animals, especially at sublevel XVIIc with a percentage of 72% (TD10-1 = 78 of 454 tooth-marked bones; XVIIc = 21/29; XVIIa = 12/28; IV = 41/142). This phenomenon may have altered the initial composition of the faunal assemblages generating a bias on specific anatomical portions. Taking into account the above-mentioned factors, bone breakage at Gran Dolina TD10-1 and Bolomor seems to correspond more to a strategy focused on marrow removal than to a repetitive exploitation of grease by crushing and/or grinding.

The intentional extraction of marrow tends to follow standardized patterns on ungulate long bones in some levels of Bolomor. Unlike other processing activities, this systematisation does not appear to respond to physical or morphological conditioning factors of the bone. According to our application of the Spearman's rank correlation test, the bone density of the anatomical portions by species and the location of percussion notches on identified skeletal elements are positively correlated (TD10-1: ρ = 0,42587, p-value = 0.01895; IV: ρ = 0,46444, p-value = 0.009721), i.e. the bones do not seem to be intentionally broken in the less dense zones (Table S4). In spite of this, there is a significant contrast of the marrow with fat availability in equids and cervids which may entail different techniques for the extraction of internal resources regardless of bone density. Binford [3] noted that the mechanical extraction of caribou marrow from metapodials was determined by the need to open up the diaphyseal tube without imbedding the marrow with bone fragments. This can be contrasted with the relatively small marrow cavity of equid metapodials, which are difficult to break and stingy in yield compared to the amount of grease held in the cancellous tissue of the thin cortex proximal and distal ends [121]. The fracture of these two taxa may be different while achieving nutritional goals, and thus, the impacts on dense *vs.* less dense zones may be fundamentally different. However, if we observe the impact distributions by taxa at level IV of Bolomor, the notch distribution pattern by skeletal element tends to be the same regardless of species. Both artiodactyls and perissodactyls show, for example, the same standardized damage on the posterior side of distal metadiaphysis in tibiae. Thus, a guide for breaking based on the bone density differences by taxa cannot be suggested at least in the cases presented here. Besides, if there existed a guide for every particular species, a high diversity in the notch distribution among the remains of the same taxa would not be registered in the case of TD10-1 (serve as an example humerus of *Cervus elaphus*) (see Table 3).

**Figure 3:**
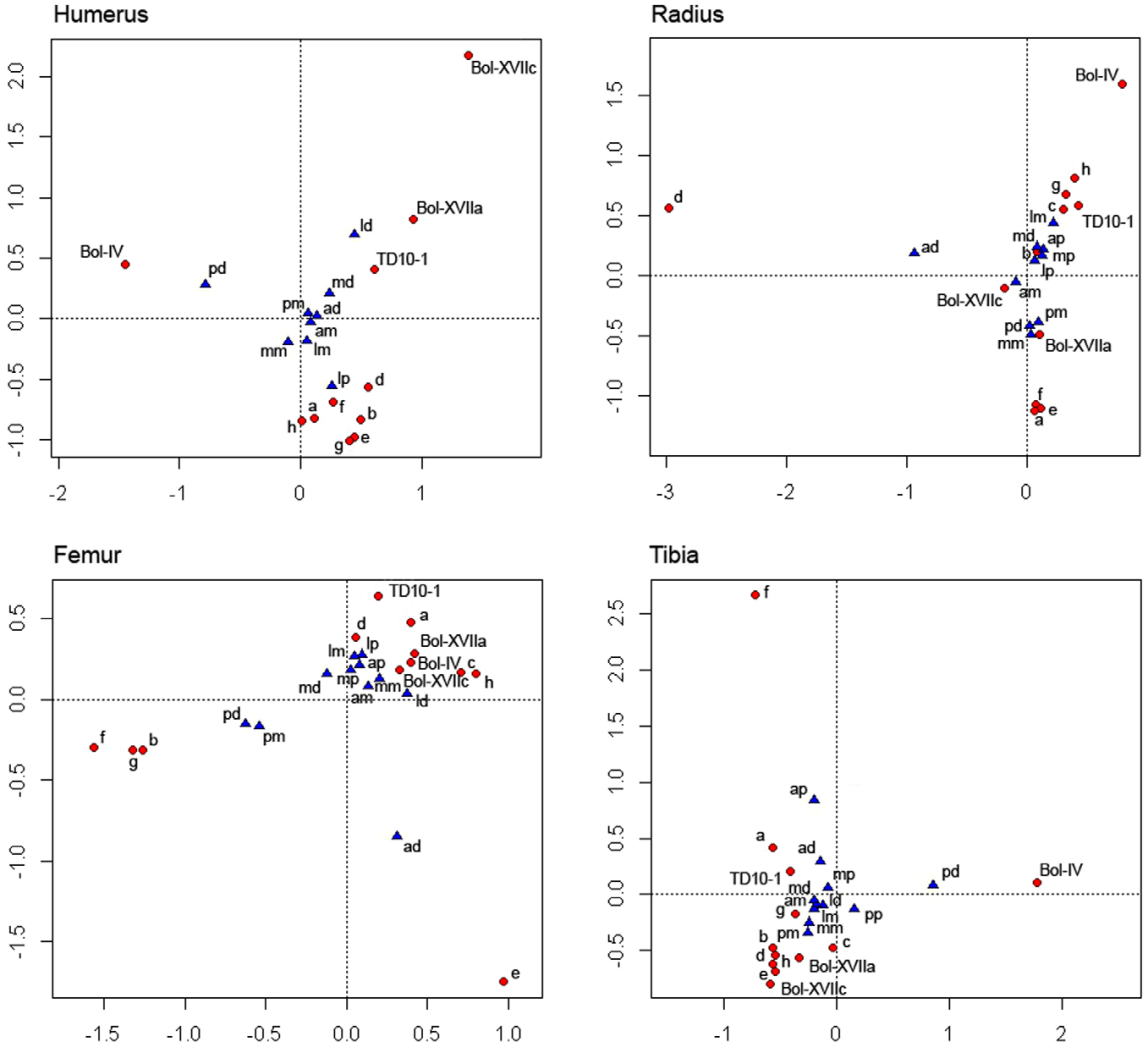
Multiple correspondence analysis showing the distances between location of notch types and the cases comprising the experimental series 1 (hammerstone percussion) and archaeological samples (data from Text S3; see Statistical methods in Text S1 for more details). Individual characters (a–h) correspond to the 8 individuals involved in the experimental series 1 (each subject is represented by an alphabetic letter). Coupled characters indicate bone region -side and portion- (e.g., ap = anterior, proximal metadiaphysis; mm = medial mid-shaft; pd = posterior, distal metadiaphysis).

**Figure 4:**
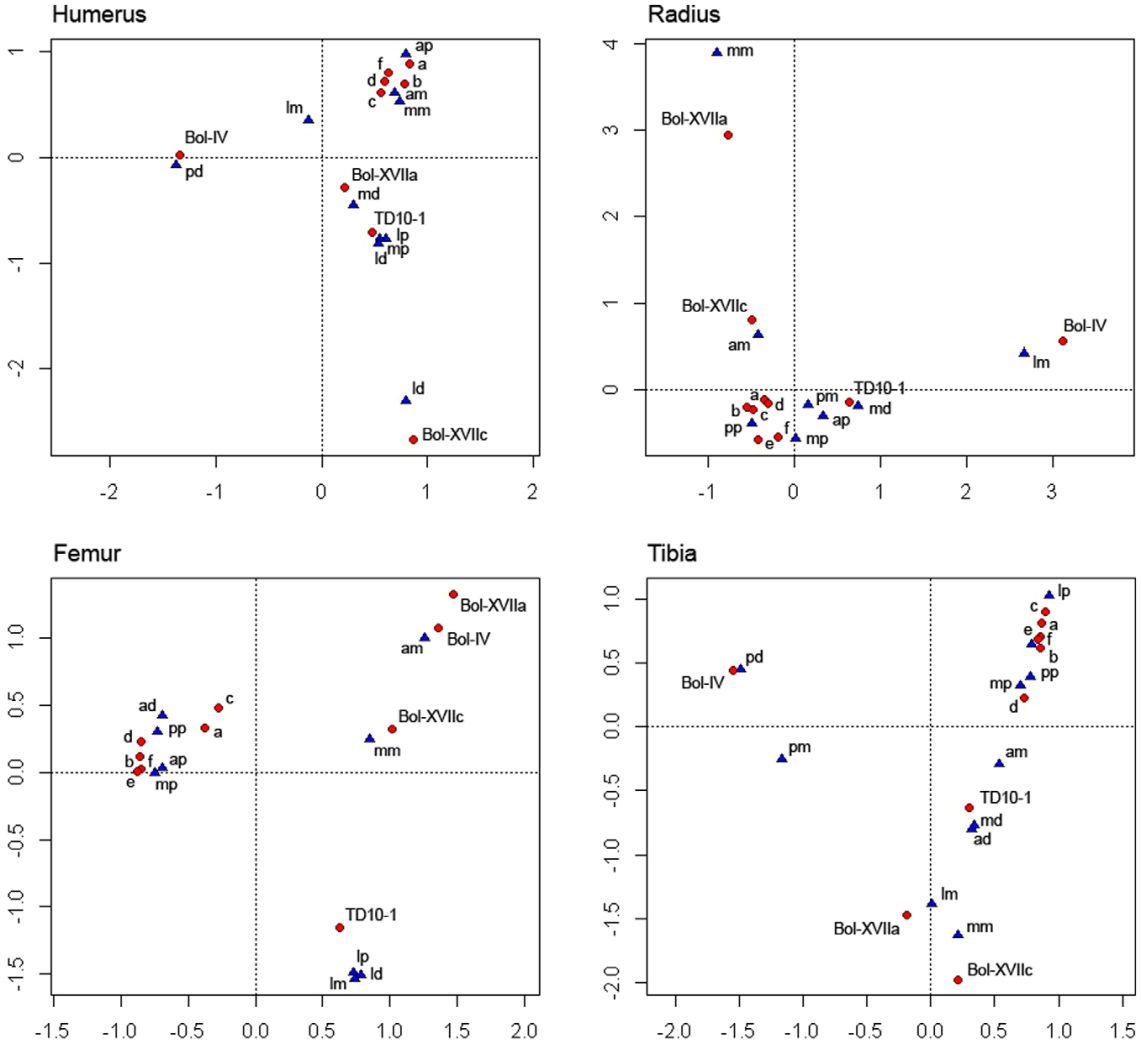
Multiple correspondence analysis showing the distances between location of notch types and the cases comprising the experimental series 2 (percussion by batting) and archaeological samples (data from Text S3; see Statistical methods in Text S1 for more details). Individual characters (a–f) correspond to the 6 individuals involved in the experimental series 2 (each subject is represented by an alphabetic letter). Coupled characters indicate bone region -side and portion- (e.g., ap = anterior, proximal metadiaphysis; mm = medial mid-shaft; pd = posterior, distal metadiaphysis).

To check other morphological conditioning factors, two experimental series were conducted under conditions of isolation, in which none of the individuals could see how bones were broken by other members participating in the experiment. Our attempt was to reproduce a context in which there was no knowledge transmission in between non-trained experimenters (Text S3). In cases where humeri were broken by hammerstone percussion (experimental series 1), the selections made by each individual experimenter concerning the same impact location may have arisen from their implementation of intuitive parameters, i.e. the medial side of humerus is flatter than the curvy lateral side and is more apt to stabilize the shaft prior to impact. Likewise, the proximal lateral shaft exposes a wider area for impact (and thinner than the distal shaft) which is ideal for bone breakage. However, humeri from level IV of Bolomor show a different preferred selection for impact, which is located on the distal posterior metadiaphysis (Text S3; Figure 3). This notch distribution pattern seems to follow counter-intuitive parameters not related to bone morphology, since humeri show a thicker cortex anterior-posteriorly [122]. But at this point, the question arises whether the use of another technique could generate similar patterns to those observed at Bolomor IV and in turn, different from those caused by hammerstone percussion. The EMT results from experimental series 2 show that notches produced when the bone is hit directly against a stone object or an anvil are patterned (p-values = 0 for all long bones). This type of bone-breaking process makes experimenters select the impact area by judging the ergonomics of the bone; i.e. the subjects tend to hold the bones by the narrower zone and hit by the wider area depending on the skeletal element. Similar reiterations in the spot location were experimentally documented by Peretto et al [123] during the processes of percussion by batting, which reassert the importance of the morphological factors when this technique is used. However, the FET outcomes display that these damage distribution patterns do not coincide with those from Bolomor IV, discarding the functional convergence in this case and suggesting that such patterns may have been behaviourally-induced (Text S3; Figure 4). This idea does not exclude the possibility that mechanically efficient patterns can be the result of acquired knowledge in bone breakage methods. Different factors may condition the human reasons that guide the processing of faunal resources, amongst which, bone morphology cannot be discarded. The archaeological problem in such cases lies in differentiating when a functional convergence phenomenon exists or when standardized damage is the result of information-transmission mechanisms. In the case of Bolomor IV, this question is cleared up, since the reiterations of impacts do not coincide with intuitive, functionally effective or potentially convergent ones.

Different forms of processing can be observed ethnographically depending on the group. Examples of standardised actions to break the bones have been observed in present-day hunter-gatherers, such as the !Kung [21] or the Nunamiut [3], [20], for example. In all cases, there are previous traditions that are fixed in the memory of the group, which are repeated through time without the need to be checked. This phenomenon could be thought of as “learning by heart”, i.e., learning something so well that it can be repeated mechanically without thinking. In the bone breakage process, these behaviours seem to cause the majority of the patterns. A noteworthy case is observed by Yellen [21] in the breakage of the kudu radius among the !Kung. The young Bushmen have learned from older individuals that the heads of the kudu radius do not contain enough marrow and therefore, this anatomical segment is treated in a different way. This conception is a subjective and unchecked judgement (inherited concept), but, nevertheless, is learned and repeated.

The consequences of a “learning by heart” procedure limit the acquisition of innovative skills without breaking customs, and demands further empirical and theoretical insights. For instance, the phenomenon of apprenticeship has been observed in the lithic industry both ethnographically [124] and at the archaeological level in several Upper Palaeolithic sites, such as Geissenklösterle [125], Gough’s Cave [126], Marsangy [127], [128] and Oldeholtwolde [129], or even in the Chatelperronian site of the Grotte du Renne at Arcy-sur-Cure [130]. However, these learning processes can be tracked almost as far back as the Middle Pleistocene. Significant examples are documented at site K of Maastricht-Belvédère [131] or at several localities of Rhenen [131], [132], where some failed lithic artefacts have been thought to be pieces created by children during their learning processes.

### Bone Breakage Patterns as an Element to Identify Occupational Dynamics

Understanding what type of occupation existed at Pleistocene sites is a complex issue, especially if we take into account that the formation processes of the archaeological levels can mix up occupational events or make them indistinguishable. Nevertheless, some elements can help us to define the main types of settlement using the faunal approach, such as the volume of archaeological material in relation to the rate of sedimentation, the degree of intervention of carnivores, species diversity and variety of procurement methods, and the hearths and spatial distribution, among others. Many of these elements cannot be individualised, since they could characterise both short and long-term occupations. For instance, continuity in specific human occupations could culminate in greater control over the territory and, therefore, greater amplitude in the exploitation of faunal resources, which would result in high taxa diversity in the archaeological record. However, we must take into account that short-term settlements can also record a wide variety of taxa. This is because they often represent stops along the way, at which the animals of the immediate surroundings are exploited without any selection criteria [133]. The accumulation of different short-term events in archaeological sites with low sedimentation rates could lead to a palimpsest characterised by species diversity. This diversity in the taxonomic profile could be similar to that observed in occupations of a longer duration. Another ambiguous element is the presence of anthropic combustion structures. For some authors, the presence of hearths is evidence of the residential character of the settlements, regardless of their duration [4], [134]–[138]. At the ethnographical level, some groups of hunter-gatherers build hearths not only at base camps, but also in places that are briefly occupied [3], [116]. In line with this evidence, the combustion structures documented at archaeological sites could represent either important occupational continuance or a short-term settlement. For this reason, their relationship with other elements becomes essential in order to determine the general dynamics of human occupations during the formation of an archaeological level. In this regard, the presence or absence of bone breakage patterns within an assemblage represents an important exception. The presence of this factor can help us to infer the existence of a cultural tradition in the area and, therefore, a pattern of relatively stable territorial occupation. In this sense, a search for breakage patterning should be considered a valid addition to discussions about human occupational dynamics.

Since members of a group tend to repeat actions, either through learning or due to guided imitation of observed actions in others [3], [13], [21], [88], the systematisation of faunal processing enables inferences to be drawn concerning the prolonged presence of a particular group in a site, or the persistence of a shared cultural tradition in the locality, which may be related indirectly to a territorial stability. On this basis, the low standardisation documented in bone breakage damage at TD10-1 could imply not only the existence of various groups in the area but also the brief character of their occupations. If a group inhabited Gran Dolina for a long time period, a higher number of repeated actions should be identifiable in the faunal record. On the other hand, a lack of standardisation could represent the intercalated occupations of different groups with different ways of processing carcasses during the formation of the assemblage (repeated short-term occupations). From this zooarchaeological perspective, it is not yet possible to establish a cultural tradition among the hominids that inhabited Gran Dolina. With this assumption, we do not state that the human groups of TD10-1 were not able to develop patterns as a result of learning, but that the general dynamic of the human occupations during the assemblage formation prevents the observation of such phenomena.

The opposite case can be observed at level IV of Bolomor, where a reiteration has been identified as sufficiently high at the quantitative level to consider systematisation when bone-marrow is intentionally extracted. This fact allows us to propose the existence of groups with the same way of proceeding, making it possible to establish a cultural tradition during the formation of this stratigraphic deposit. But, unlike TD10-1, the existence of highly standardised bone breakage patterns does not solve the question related to the durability of the settlements. In the level IV case, which can also be extended to the XVIIc sublevel, the presence of patterns raises at least two possibilities: 1) the cave was inhabited by human groups with shared cultural traditions during repeated but short-time periods or; 2) the cave was used by human groups with shared cultural traditions during long-time periods. On this basis, the archaeological line that differentiates the types of settlement is diffuse and therefore, we must resort to other elements or disciplines that can complement the data provided by bone breakage patterns. From this approach, many different processes related to both human activity and the action of carnivores or raptors occur during the formation of the archaeological assemblages. The actions that these non-human predators generate on the faunal set, either by modifying or adding elements (e.g., coprolites, transporting specific skeletal elements), are an essential tool for inferring the existence of periods of human abandonment of the site. The shortage of carnivore damage at level IV (0.5%; NISP = 142; Total NISP = 25323), together with the presence of standardised bone breakage patterns, may be used to back up the hypothesis related to several long-term human occupations during the formation of this level. In contrast, at TD10-1, the presence of damage is higher (4%; NISP = 454; Total NISP = 11081). In addition, some characteristic elements of carnivore dens, such as digested bones, pitting or diaphyseal cylinders, have been recovered. These data allow us to suggest the existence of intermittent periods of human occupation alternating with brief intrusions of carnivores during the formation of TD10-1. On this basis, the low sedimentation rates at the bottom of the sublevel may have helped the overlapping of several types of occupations, producing accumulations that are apparently uniform at the archaeological level [139].

Both TD10-1 and Bolomor level IV seem to correspond to extremes within the occupational dynamics. However, between a short-term human occupation and a long one, there exists a wide range of potential settlement types [140]–[146]. An example of intermediate occupations could be suggested at sublevel XVIIa. Although in this assemblage some repetitions have been registered, variability is one of the main characteristics in the extraction of the internal resources from the bones. This variability does not reach the degree of diversity identified at TD10-1 or the standardisation documented at level IV or sublevel XVIIc. Therefore, it is possible that XVIIa shows an intermediate dynamic or that it responds to an accumulation mainly generated by short-term human occupations with the odd event of certain stability, although not excessively long. At this point, we must note that our interpretations attempt a general theoretical explanation of the nature of the assemblages and it is possible that within the dynamics outlined, other sporadic events may exist, causing exceptions and distortions. In addition, both the XVII (a/c) level and especially the XI level contain relatively few bones with percussion notches and, therefore, the breakage patterns should be interpreted with caution. It must be noted that not all fragments can be attributed to a skeletal element, and this is a very common problem when dealing with bone splinters. Unidentified fragments most likely derive from fore and hind limb shafts, but their recognisability is a critical problem [20].

The case of small prey also needs mentioning, because its breakage patterns appear to constitute an exception. We consider small prey here to be species of less than 10 kg (rabbits, hares, birds, etc.). Within this rubric, small game at Gran Dolina TD10-1 and Bolomor does not present the same diagnostic elements of breakage as those observed on ungulate remains; i.e. small prey bones show no diagnostic features of fracturing by active or passive percussion such as percussion pits, percussion notches, impact flakes or counterblows. Nevertheless, standardized morphotypes are observed on their bones, overall on the fore and hind limbs of rabbits. These patterns are the result of the isolation of the epiphyses or epiphyses with part of the metadiaphyses in stylopodials and zeugopodials. This almost systematic process of separation gives rise to a large number of diaphyseal fragments that include from one to all four sides of the bone (diaphyseal cylinders).These well-established morphotypes are generated when the limb bones are broken using the teeth, hands or stone hammers on epiphyses or metadiaphyses. At both sites, shaft cylinders have been recovered together with a high proportion of extremities, which could be related to the use of the bite and/or flexion or the combination of both actions, rather than hammering. For this reason, the zone methods used to analyse impacts on large mammal bones are not appropriate for small prey remains. In Bolomor, the same morphotypes are repeated in a more or less standardised way throughout the whole sequence, irrespective of the type and intensity of the occupation. Based on this, tackling the occupational dynamics from the search for patterns in these animals is not possible. This phenomenon could represent *a priori* a theoretical contradiction with our proposition that standardised processing sequences may form a characteristic feature of a group. However, we must take into account that significant differences exist between ungulates and small prey that enable the individualisation of their sequences of exploitation and distinctions to be made in their handling [88]. Therefore, the same criteria cannot be applied or assumed when establishing patterns of action on these animals. Distinguishing characteristics, such as the size and the use of hands or teeth as the main tool for the immediate consumption seem to condition the appearance of these standardised morphotypes on the bones, regardless of the site location or chronology. The conditioning factors of these animals allow the systematization to be recorded in numerous periods and sites, such as at level 4 of the French Mousterian site of Canalettes [69], [70], at different sites of the European Upper Palaeolithic (e.g.,[72], [147]–[155]) and even among some present-day hunter-gatherer groups, such as the Aché of Paraguay [156].

The transmission of knowledge as an explanation for the standardization of the archaeological record can be identified in other behavioural domains. In fact, it is worth analyzing whether a correlation exists between standardization in bone breakage patterns and, for example, knapping strategies and tool manufacturing. In lithic assemblages, standardized morphological patterns are well-known since the appearance of handaxes in the Early Pleistocene sites. It has been suggested that Acheulean assemblages indicate the existence of well-structured strategies for knowledge transfer between generations [157]–[159], which would explain the wide spatial and temporal distribution of handaxes. Some ethnographic references suggest that manufacturing bifacial morphologies similar to Acheulean handaxes require long and complex learning processes [160]. The chronological span that includes the assemblages analyzed in this paper coincides with the emergence in Europe of the Levallois method [161], which is based on a well-defined volumetric conception of the core oriented to predetermine blank shape and/or size. This volumetric design results in a great standardization of core morphology, which would be difficult to explain if knowledge transfer processes were absent from groups. The preconceived character of this knapping method clearly distinguishes the Levallois assemblages from those associated with expedient strategies, which are simply aimed at reducing the core in a recurrent way, without a predetermination of the products. Therefore, the ability to adjust the technical behaviour to transmitted normative patterns seems to be fully acquired in the late Middle Pleistocene. However, when we talk about handaxes and Levallois cores we are referring to major technological categories that are represented in the archaeological record for hundreds of thousands of years throughout much of the Old World. We need a more fine-grained analysis of technical variability to identify more discrete spatial and temporal units, showing standardized patterns specific to certain regions or sites, to approach social entities such as those inferred from level IV of Bolomor Cave. Identification of small-scale standardized patterns in both subsistence and technology opens an interesting avenue for future researches.

### Conclusions

Bone damage generated during the extraction of external and internal resources can be used to assess the existence of systematic activities or standardised processes among human groups. In the case of the TD10-1 sublevel from Gran Dolina and the XVII, XI and IV levels from Bolomor Cave, the location, disposition and distribution of modifications in terms of anatomical area and region (portion and side) have been used to observe possible human reiterations on faunal assemblages. The development of some butchery activities, such as defleshing, appears to be conditioned by several factors (e.g., method of acquisition, the animal’s anatomy, prey size, the experience of the butcher, site functionality, seasonality, ground features and/or available human technology) and therefore, hinder the archaeological identification of certain cultural processes within the processing sequence. A different case appears to arise in bone-marrow extraction through the intentional breakage of the bones that make up the analysed sets. Statistical tests used in this study show no correlation between the less dense areas and the localisation of the impacts, i.e., there appear to be no guidelines based on the density of certain areas of the skeletal element when fracturing the bones. Our EMT results from experimental series show that the morphological factors do not seem to condition the repeated selection of impacts for breaking open the shafts. Level IV of Bolomor gives an example of a counter-intuitive preferred selection for impacts. The repetition of impact points appears to be the result of more complex processes related to the transmission of intergenerational information within each group (cultural patterns). On this basis, the hominids of the European Middle Pleistocene appear to be capable of developing learning mechanisms that culminated in their own cultural traditions, which are different to those developed by other communities, suggesting the existence of a certain group or territorial entity. The existence of these traditions within the groups and their reflection at the archaeological level in standardised patterns can significantly contribute to interpreting the occupational dynamics in the territory during the formation of the assemblages. Thus, the low standardisation documented at TD10-1 could be the result of the presence of various different groups with different ways of processing the carcasses. The opposite case can be observed at level IV of Bolomor, where a reiteration has been identified that is high enough at the quantitative level to consider systematisation in marrow removal. This fact could suggest the presence of relatively lengthy occupations that would share this behaviour, making it possible to establish a cultural tradition during the formation of the set. Thus, a different debate about the significance of patterning is possible from the faunal assemblage. The identification of these intra-group information transmission processes allows us to suggest the existence of a high social component and perhaps specific socialisation processes in which learning represents an important element for the continuity over time of the human groups of these chronologies. This social cohesion would also be essential for the successful development of several hunting strategies, particularly those involving large ungulates or those entailing the practice of complex techniques. Finally, and as a future prospect, the methodology developed here to assess occupational dynamics should be set out within a broader general context, at both geographical and chronological level. In the same way, the interpretations of this paper must be viewed as a starting point. These ideas should be contrasted at other sites and with other disciplines with the aim of determining different dynamics and, therefore, being able to assess the different subsistence strategies developed by the human groups in other spaces and environments, contributing to the knowledge of the ways of life of the hominids of the European Middle Pleistocene.

## Supporting Information

Table S1 **Results from the Exact Multinomial Test (EMT) for sublevel TD10-1 of Gran Dolina and level IV of Bolomor Cave.** Starting from the ab-initio hypothesis that impacts should be uniformly distributed across bone parts, probabilities in the tested null-model have been adjusted. See Text S1 or http://rgm2.lab.nig.ac.jp/RGM2/func.php?rd_id=EMT:multinomial.test for more details.
(XLS)

Table S2 **NISP with cut-marks, location, morphology and performed activity by skeletal elements, taxa and weight size category from TD10-1.** Abbreviations: Cm = Cut-marks; Inc = incisions; Saw = sawing marks; Scr = Scrape marks; Sk = Skinning; Df = Defleshing; Da = Disarticulation; Dm = Dismembering; Vr = viscera removal; Pr = Periosteum removal; Tr = Tendon removal.
(XLS)

Table S3 **NISP with cut-marks, location, morphology and performed activity by skeletal elements, taxa and weight size category from the archaeological.** Abbreviations: Cm = Cut-marks; Inc = incisions; Saw = sawing marks; Scr = Scrape marks; Sk = Skinning; Df = Defleshing; Da = Disarticulation; Dm = Dismembering; Vr = viscera removal; Pr = Periosteum removal; Tr = Tendon removal.
(XLS)

Table S4 **Bone density data estimated by Lyman** [**22**] **and Lam et** **al.** [**47**] **, NISP with percussion notches and correlation coefficient by means Spearman’s** ***rho*** **and Kendall’s** ***tau*** **from TD10-1 and Level IV.**
(XLS)

Text S1 **Statistical methods used to approach pattering in the archaeological and experimental samples.**
(DOC)

Text S2 **Results from the Exact Multinomial Test (EMT) and Fisheŕs exact test (FET) applied to each site and level to approach the cut-mark distribution.**
(DOC)

Text S3 **Experimental series and its comparison with the archaeological cases of Gran Dolina TD10-1 and Bolomor Cave.**
(DOC)
