# Supplementary material for: Learning by Heart: Cultural Patterns in the Faunal Processing Sequence during the Middle Pleistocene
Source: PLoS One. 2013 Feb 20;8(2):e55863. doi: 10.1371/journal.pone.0055863 (PMC3577810; doi:10.1371/journal.pone.0055863)
Supplement: Text S1 — Statistical methods used to approach pattering in the archaeological and experimental samples. (DOC) [file pone.0055863.s005.doc]

**Text S1. Statistical methods used to approach pattering in the archaeological and experimental samples.**

Patterning in the samples was statistically approached by using the Exact Multinomial Test (EMT), which is a goodness-of-fit test for discrete data. EMT provides an estimate of the probability that values in a vector occur patterned or randomly, under the assumption of an ab-initio model. Such model is created by providing each category with a theoretical probability that an item can fall into it. The resulting p-value yielded by the EMT is an estimate of the probability that an observed sample fits with the ab-initio model, since this latter also contains a description of the degree of randomness in it. EMT calculates the exact probabilities of all possible outcomes of the contingency table that mixes the number of cases and groups. In the present study, this was performed with the R library “EMT” and the function “multinomial.test”. In the present work, a Monte Carlo approach was used, because given the characteristics of the vectors used, the computation of the numerical calculation provided vectors of several Gigabytes (GB). To enhance calculation, a Workstation (Mountain) was used, which was provided with a RAM memory of 16 GB. The Monte Carlo approach simulates withdrawal of *n* samples of size *n* from the hypothetical distribution. The number of samples withdrawn from each calculation in the present analysis was 4.000.000. The p-value provided by this approach is the result of the addition of the relative frequencies of occurrence of outcomes which were less frequent than the outcome documented in the ab-initio model.

For the present analyses, the ab-initio model was defined as follows. Each bone was divided into five different sections: proximal epiphysis, proximal metadiaphysis, mid-shaft, distal metadiaphysis and distal epiphysis. The assumption that each of these sections would be equally likely to receive an impact is biased by the perception of butchers that the area to be impacted is the shaft. For this reason, epiphyseal sections were discarded from the ab-initio model. This was elaborated considering only proximal metadiaphysis, mid-shaft, and distal metadiaphysis. A similar case can be observed in the cut-mark distribution. The preliminary tests provided significant patterning because there is a paucity of cut-marks on the long bone ends and most of them cluster on shafts. For this reason, this potential bias might be masking a real cut-mark patterning. This prompted us to dispense with ends and focus on cut-mark distribution on long bone shafts. Each section has four sides (anterior or cranial, posterior or caudal, medial and lateral) which are initially equally likely to receive an anthropogenic damage. The ab-initio model, thus, considered equal probabilities of impact on 3 sections x 4 sides, with a resulting model of 12 parameters. Randomness was defined as anthropogenic damage producing impact notches and cut-marks evenly distributed among the 12 parameters. A patterned model was defined as notches or cut-marks showing some statistically significant occurrence on any of the parameters of the original parameter set.

After documenting patterning and randomness in the experimental sets and on two archaeological assemblages (TD-10-1 and Bolomor level IV), differences within each group and inter-groups were statistically approached (see Text S2 for cut-marks in archaeological samples and Text S3 for percussion notches in experimental and archaeological samples).

For testing differences of categorical groups with factors in contingency tables, Fisher´s exact test (FET) was selected over χ^2^, because it is more accurate when sample sizes are small. χ^2^ yields an approximation significance value because the statistic it calculates is an approximation to the theoretical distribution. FET, in contrast provides a more accurate estimate because the significance of the deviation from the null hypothesis can be calculated exactly. χ^2^ is inadequate also when the data are very unequally distributed among the cells of a table with expected values being frequently underestimated. The use of χ^2^ is not adequate when values in any of the table cells are below 5 or 10. In the contingency tables used for the present study, this characteristic was common in all the samples, both the experimental and the archaeological ones. For this reason FET was preferred in the analysis.

FET was also used to discern if patterning was documented when comparing occurrences of anthropogenic damage on bone sections. In the experimental sample, patterning was expected if all the experimenters broke bones similarly (see Text S3). In the archaeological sample, notches (Text S3) and cut-marks (Text S2) were also compared among sites and within Bolomor IV more specifically, to detect a preferential location of notches or a more random distribution.

These tests were graphically complemented with a correspondence analysis, which showed the distances between location of notch types and the cases comprising the experimental and archaeological samples (see Text S3). An asymmetric graph was selected, with rows in the principal coordinates and columns providing the standardized residuals. The “ca” R library was selected for this purpose.
