# Supplementary material for: Learning by Heart: Cultural Patterns in the Faunal Processing Sequence during the Middle Pleistocene
Source: PLoS One. 2013 Feb 20;8(2):e55863. doi: 10.1371/journal.pone.0055863 (PMC3577810; doi:10.1371/journal.pone.0055863)
Supplement: Text S2 — Results from the Exact Multinomial Test (EMT) and Fisheŕs exact test (FET) applied to each site and level to approach the cut-mark distribution. (DOC) [file pone.0055863.s006.doc]

**Text S2. Results from the Exact Multinomial Test (EMT) and Fisher´s exact test (FET) applied to each site and level to approach the cut-mark distribution.**

Given the different anatomical structure of macrofaunal mammals and those of leporids and birds, the patterning statistically analysed was restricted to the macromammals provided in the Table 1, 4 and 5 (Cervidae, Equidae and Bovidae). To make comparisons similar to the study of long bone breakage and notch distribution (see Text S3), cut-marks were studied on long bones (except on ulnae). The preliminary tests provided significant (p-values=0.000) patterning in cut-mark distribution because there is a paucity of cut-marks on the long bone ends and most of them cluster on shafts. For this reason, this potential bias might be masking a real cut-mark patterning. This prompted us to dispense with ends and focus on cut-mark distribution on long bone shafts.

Twelve shaft bone sections were differentiated (proximal, mid-shaft and distal multiplied by the four orientations: anterior or cranial, posterior or caudal, lateral and medial).

The multinomial test applied to each site and level showed the following results:

|  | TD10-1 | Bolomor XVIIc | Bolomor XVIIa | Bolomor XI | Bolomor IV |
| --- | --- | --- | --- | --- | --- |
| Humerus | **0.000** | 0.655 | 0.915 | 0.213 | **0.000** |
| Radius | **0.501** | 0.484 | 0.345 | 0.877 | **0.000** |
| Femur | **0.000** | 0.610 | 0.461 | 0.390 | **0.000** |
| Tibia | **0.000** | **0.0491** | 0.991 | 0.953 | **0.000** |
| Metacarpal | 0.711 | - | - | - | **0.000** |
| Metatarsal | 0.818 | 0.121 | **0.000** | - | **0.000** |

The p values (p-value <0.05 indicates that the observed model differs in a more patterned way from the ab-initio model) show that for most long bones from Bolomor XVIIc, XVIIa and IX there is no indication of patterning, in contrast with the cut-mark distribution on long bones from TD10-1 and Bolomor IV. Whereas this may be suggesting of random placement of cut-marks on the Bolomor levels (other than Bolomor IV) it actually has more to do with sample size. The number of cut-marks reported for Bolomor XVIIc,a and IX is substantially smaller than those reported for TD10-1 and Bolomor IV. Given that the EMT tests are very conservative, when sample size is small no pattern is detected. However, the larger sample of cut-marks for the other two sites indicates that pattering exist, in some cases (such as at Bolomor IV) for all long bones. The reason for this is that, despite the bias in preservation of various sections (properly taken into account in the ab-initio model), cut-marks cluster preferably on mid-shafts instead of on metadiaphyseal sections. This pattern is the one that the multinomial tests are detecting.

A FET comparing the cut-mark distribution between TD10 and Bolomor IV on all long bones yielded the following result:

| Humerus | 0.020 |
| --- | --- |
| Radius | 0.000 |
| Femur | 0.000 |
| Tibia | 0.040 |
| Metacarpal | 0.006 |
| Metatarsal | 0.000 |

In all long bone significant differences were detected comparing the patterned distribution of cut-marks.
