# Supplementary material for: Learning by Heart: Cultural Patterns in the Faunal Processing Sequence during the Middle Pleistocene
Source: PLoS One. 2013 Feb 20;8(2):e55863. doi: 10.1371/journal.pone.0055863 (PMC3577810; doi:10.1371/journal.pone.0055863)
Supplement: Text S3 — Experimental series and its comparison with the archaeological cases of Gran Dolina TD10-1 and Bolomor Cave. (DOC) [file pone.0055863.s007.doc]

**Text S3. Experimental series and its comparison with the archaeological cases of Gran Dolina TD10-1 and Bolomor Cave**

In order to check the possible existence of guides based on the physical or morphological characteristics of limb bones during the breakage activities, two experimental series was carried out. Our objective was to compare the results from these experiments with the data extracted from the previously studied Pleistocene assemblages. These experiments will allow us to assess whether the patterns observed at archaeological level respond to an intuitive processing applied repeatedly according to the skeletal element morphology or on the contrary, respond to counter-intuitive patterns.

**1. Experimental series 1**

*1.1 Experimental development and materials*

The experimental series presented here involved 8 individuals (16-40 years old) with no experience related to bone breakage in fresh state (non-trained butchers). Each experimenter fractured 4 femora, 4 radii-ulnae, 4 humeri and 4 tibiae from adult cows[[1]](#footnote-2) (MNE total = 128) with the objective of extracting the bone-marrow in optimal conditions. The series was conducted under conditions of isolation, in which none of the individuals could see how bones were broken by other members participating in the experiment. In order to fracture the skeletal elements, experimenters used two techniques: 1) active percussion (percussion by batting) and 2) passive percussion (hammerstone percussion) [1, 2, 3]. Active percussion was produced when the bone was hit directly against an object (stone) and passive percussion was caused when the bone was held on the ground or on an object that acted as an anvil and was hit with an instrument made of stone. The technique mainly used during the experimental series was passive percussion (hammerstone percussion). All of the individuals broke the skeletal elements by means of this technique with the help of stone hammers (quartzite pebbles with a maximum length of 25 cm and a maximum weight of 4 kg), except for the 1st individual, who fractured one radius by batting (active percussion) and for the 4th individual, who placed systematically the bones between anvils and threw a stone of 30-40 cm in diameter in each case. All of the bone fragments resulting from experimental series were recovered and individualized according to individual and skeletal element. Surface damage caused during the bone breakage, especially percussion notches and attempts, was registered and summarized in the Figure 1.


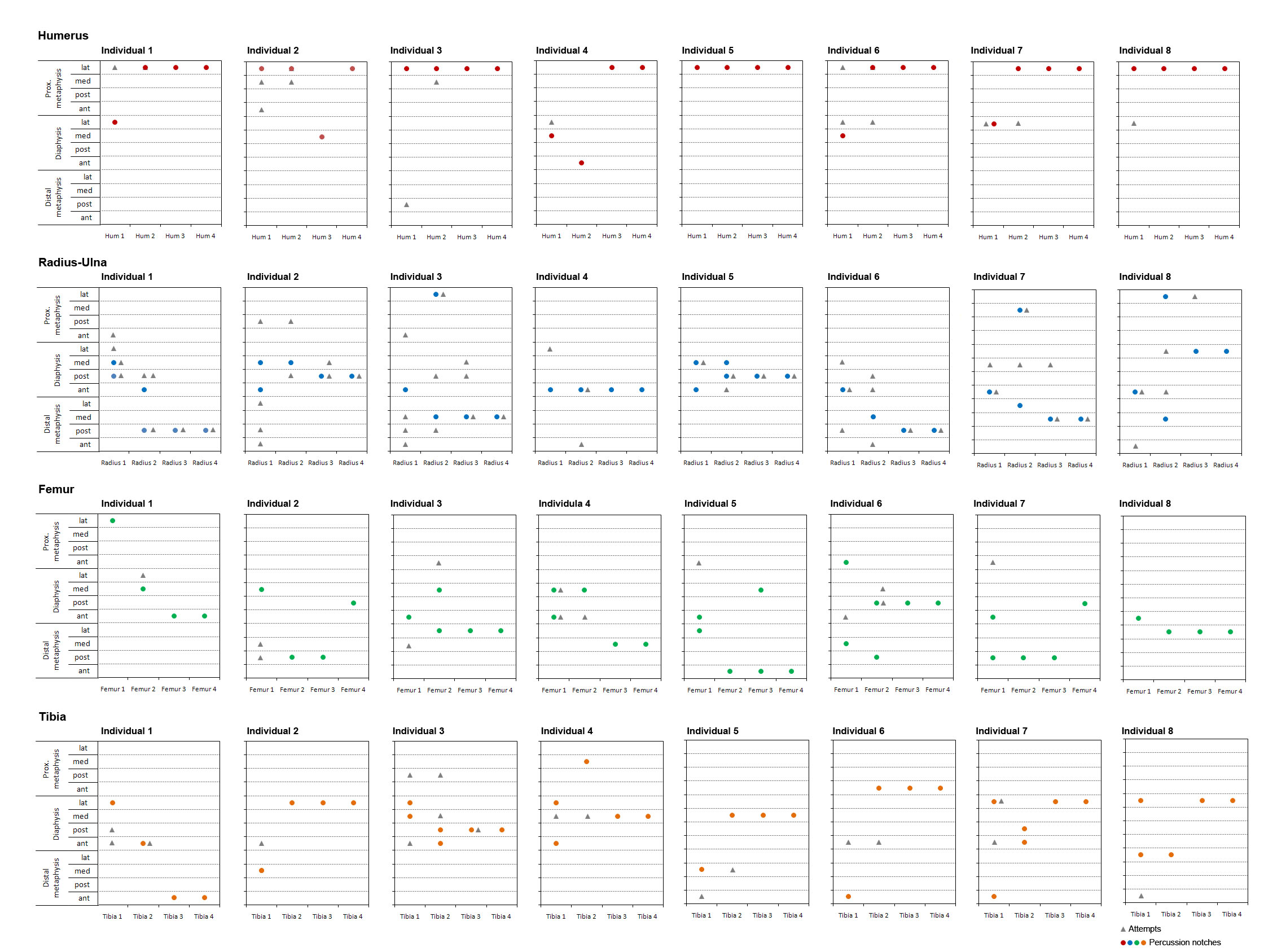
Figure 1. Summary of attempts and percussion notches according to skeletal elements and individuals during the development of the Experimental series 1.

*1.2 Results*

The EMT shows that the Bolomor IV long bone collection shows a distribution of notches that seems patterned. See this table showing the p-values (p-value <0.05 indicates that the observed model differs in a more patterned way from the ab-initio model):

| Humerus |  | >0.000 |
| --- | --- | --- |
| Radius |  | 0.043 |
| Femur |  | 0.010 |
| Tibia |  | >0.000 |

The pattern is specifically noticeable in the humerus and tibia but barely so in the radius and femur.

When checking for the number of bone portions recovered for each of our 12 parameters, we observe that Bolomor IV presents a sampling bias that is especially significant for radius and femur. In order to analyze the effect of such a bias on our study, we have recalculated the EMT with a modified ab-initio model that accounts for the different recovered frequencies. As can be seen in the table below, now the pattern is also significant for femur.

| Humerus |  | >0.000 |
| --- | --- | --- |
| Radius |  | 0.025 |
| Femur |  | 0.0001 |
| Tibia |  | >0.000 |

An interesting observation yielded by EMT was the discovery that the experimental sample *also* showed some patterning. The occurrence of notches in all long bones did not seem to follow a completely random distribution, with some sections and sides more likely to contain notches than others. These are the p-values of the EMT:

|  | experiments |
| --- | --- |
| Humerus | >0.000 |
| Radius | >0.000 |
| Femur | >0.000 |
| Tibia | >0.000 |

This clearly indicates that untrained butchers select some parts of the bones more than others to impart their blows. However, the question remains on whether all select the same parts or not.

A FET study shows that there are no significant differences on the location of impact by the 8 experimenters on the humerus (and the resulting location of notches) but each of them selected different locations for breaking open the other meaty long bones, as the p-values indicate:

| Humerus | 0.132 |
| --- | --- |
| Radius | >0.000 |
| Femur | >0.000 |
| Tibia | >0.000 |

Therefore, there is a common pattern in how the experimenters selected to break the humerus, which may be the result of the ergonomics of bone breakage given the humeral morphology, since no communication existed among the experimenters. Notches on the other long bones varied according to the experimenter.

When comparing the experimental sample, reproducing a context in which there was no knowledge transmission among the bone-breakers, to the archaeological sample, some interesting results can be observed. The following table shows the FET p-values:

|  | All sites | BOL-IV |
| --- | --- | --- |
| Humerus | >0.000 | >0.000 |
| Radius | 0.021 | 0.066 |
| Femur | 0.110 | 0.892 |
| Tibia | 0.000 | >0.000 |

If comparing the experiments to the archaeological sample (comprising Bolomor IV, XVIIa, XVIIc and TD-10-1), it can be noticed that hominids were breaking femora like the untrained experimental butchers. Significant differences were detected in how the other three meaty bones were broken. Given that Bolomor IV had a large sample of notches and is presented in this work as a potential example of knowledge-mediated bone breakage, if the experimental sample is compared to this archaeological site alone, significant differences in notch location are documented on the humerus and tibia. However, radii and femora were broken similar to the bone assemblage broken by the experimental butchers.

The graphical representation of the contingency tables (via correspondence analysis) for each long meaty bone shows the same results in more detail.

Figure 2


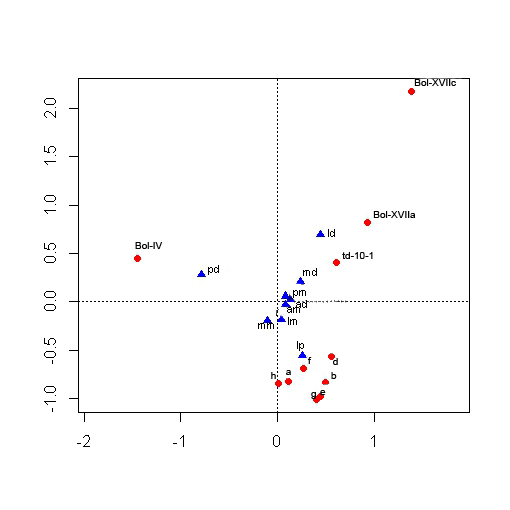


Figures show a two-dimension solution explaining 88% of the inertia. In the Figure 2, it can be seen how the experimental sample for humeri clusters low on both dimensions (lower part of the image), with each experimenter represented by an alphabetic letter and mostly influenced by impact notches on the lateral side of the proximal metadiaphysis. It also shows that all archaeological sites cluster separately from the experimental sample, indicating that TD-10-1 and Bolomor XVIIa had a more diverse location of notches and Bolomor XVIIc and IV had a core specialized occurrence of notches. A correspondence analysis on the notch location for radii (Figure 3) shows a different picture.

Figure 3


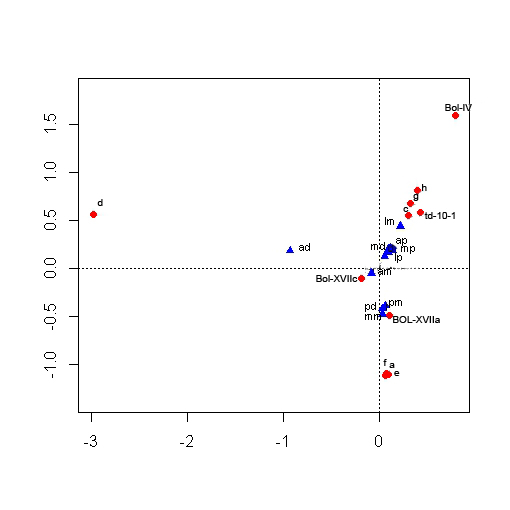


In Figure 3, all sites cluster within the area of the untrained experimental butchers with experimenter D being an outlier and Bolomor IV clearly occurring separately, but still within the area of influence of the experimental sample, as detected when comparing the experimental sample and the Bolomor IV sample via FET.

The situation with the femur is more straightforward (Figure 4). Here, as indicated by the FET, archaeological and experimental sample are statistically undifferentiated.

Figure 4


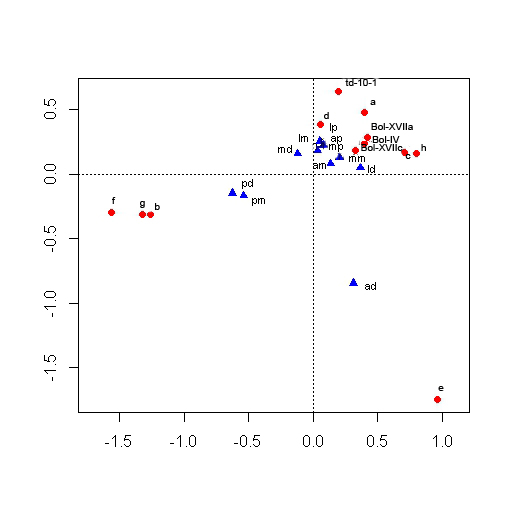


In Figure 4 all the archaeological samples fall well within the area of inertia determined by the experimental samples.

Figure 5


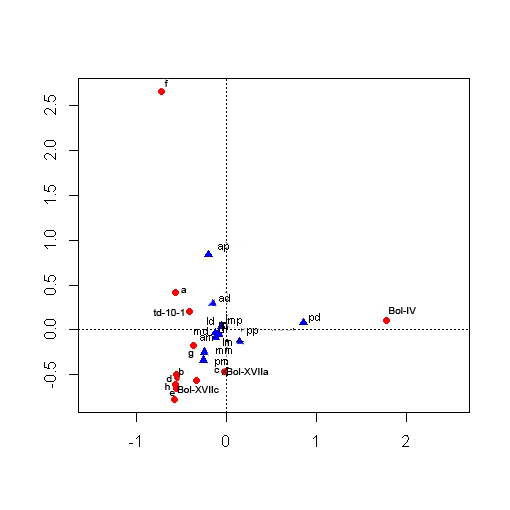


Figure 5 shows a correspondence analysis on notch location on the tibia, where although significant differences were detected when applying a FET, it was because of the weight of the Bolomor IV assemblage, which clearly shows separated from the other sites and the experimental sample.

*1.3. Discussion and conclusions*

There is certainly a pattern in Bolomor IV in the selection of section and sides where impact blows have generated impact notches. This pattern, though, is statistically detected on humeri and tibiae. It could be argued that non-trained butchers also generated patterned bone breakage, as documented in the fact that each experimenter preferred to impact each bone in certain locations (EMT results), but such pattern differs in between experimenters. The humerus is the sole exception. Here, and independently, each experimenter has selected the same impact location on most occasions. However, this selection can be explained in part because the medial side of humeri is flatter than the curvy lateral side and is more apt to stabilize the shaft prior to impact. Likewise, the proximal lateral shaft exposes a wider area for impact (and thinner than the distal shaft) which is ideal for bone breakage. Selection of this spot is rather intuitive. However, Bolomor IV shows a preferred selection for impact on the distal metadiaphysis which is counter-intuitive, since this area is thick. Humeri show a thicker cortex anterior-posteriorly [4]. The repeated selection of this spot for breaking open the shaft must have been behaviourally-mediated. Something similar could be argued about the selection at Bolomor IV of the distal posterior side of the tibia, which exhibits a significantly thicker cortical section than the remainder of the shaft. Both elements support that bone breakage was counter-intuitively applied repeatedly on these sections because it may have been part of a learned behavioural repertoire. This would add support to the integrity of the site (the same agent/s) may have been responsible for bone breakage and hence, butchery. The only argument that demands caution about applying this to the butchery of the complete animal is that no pattern has been detected on radii and femora at Bolomor IV. However, this phenomenon must not be taken into account because the sample for these skeletal elements is too small, circumstance that impedes the observation of possible patterns statistically. One could argue that if bone breakage is indeed a learned behaviour, and therefore that there are acquired ways of how to select the bone spots where impact takes place, this should be observable on *all* long bones.

**2. Experimental series 2**

*2.1 Experimental development and materials*

Individuals involved in the Experimental series 1 mainly used the hammerstone percussion. The preferred use of this technique allows us to have a suite of features to identify conditioning factors depending on each skeletal element. In order to complete experimentally the documentation of the breakage methods, a new series with the least used methods in the previous experiment was carried out. Following the techniques described by Peretto et al. [2], the Experimental series 2 was focused on the percussion by batting (active percussion); i.e. when the bone is hit directly against a stone object to be broken. This series was developed under the same variables and applying the same criteria used in the Experimental series 1. The main difference lies in the fact that the non-trained butchers were apprise of the technique to be used, but they were not instructed as to a determined way of breaking the bone.

The experimental series 2 involved 6 individuals (25-40 years old) with no experience related to this type of method. Each experimenter fractured 4 femora, 4 radii-ulnae, 4 humeri and 4 tibiae from adult cows[[2]](#footnote-3) (MNE total = 96) with the objective of extracting the bone-marrow in optimal conditions. This series was conducted under conditions of isolation, in which none of the individuals could see how bones were broken by other members participating in the experiment. Our attempt was to reproduce a context in which there was no knowledge transmission among the bone-breakers. All of the individuals hit the skeletal elements against a limestone anvil of blunt edges (62 cm length, 45 cm width and 40 cm thickness). All of the experimenters held the bones by the narrower zone and hit them by the wider area following intuitive parameters. This phenomenon was clearly observed on the radius-ulna, tibia and humerus, which were preferably hit and fractured by their proximal metadiaphyses. The time spent to fracture the bones depended on the physical characteristics of the subjects and the morphology of the different skeletal elements. Stronger or more corpulent individuals (80-90 kg) took less than 20 seconds to break open the bones, while the other ones (< 60 kg) spent more than 2 minutes since the first blow. Tibia was the easier element to fracture regardless of the individual features. Like in the experimental series 1, all of the resulting bone fragments were collected and individualized according to subject and skeletal element. Surface damage caused during the bone breakage, especially percussion notches and attempts, was documented and summarized in the Figure 6.

**
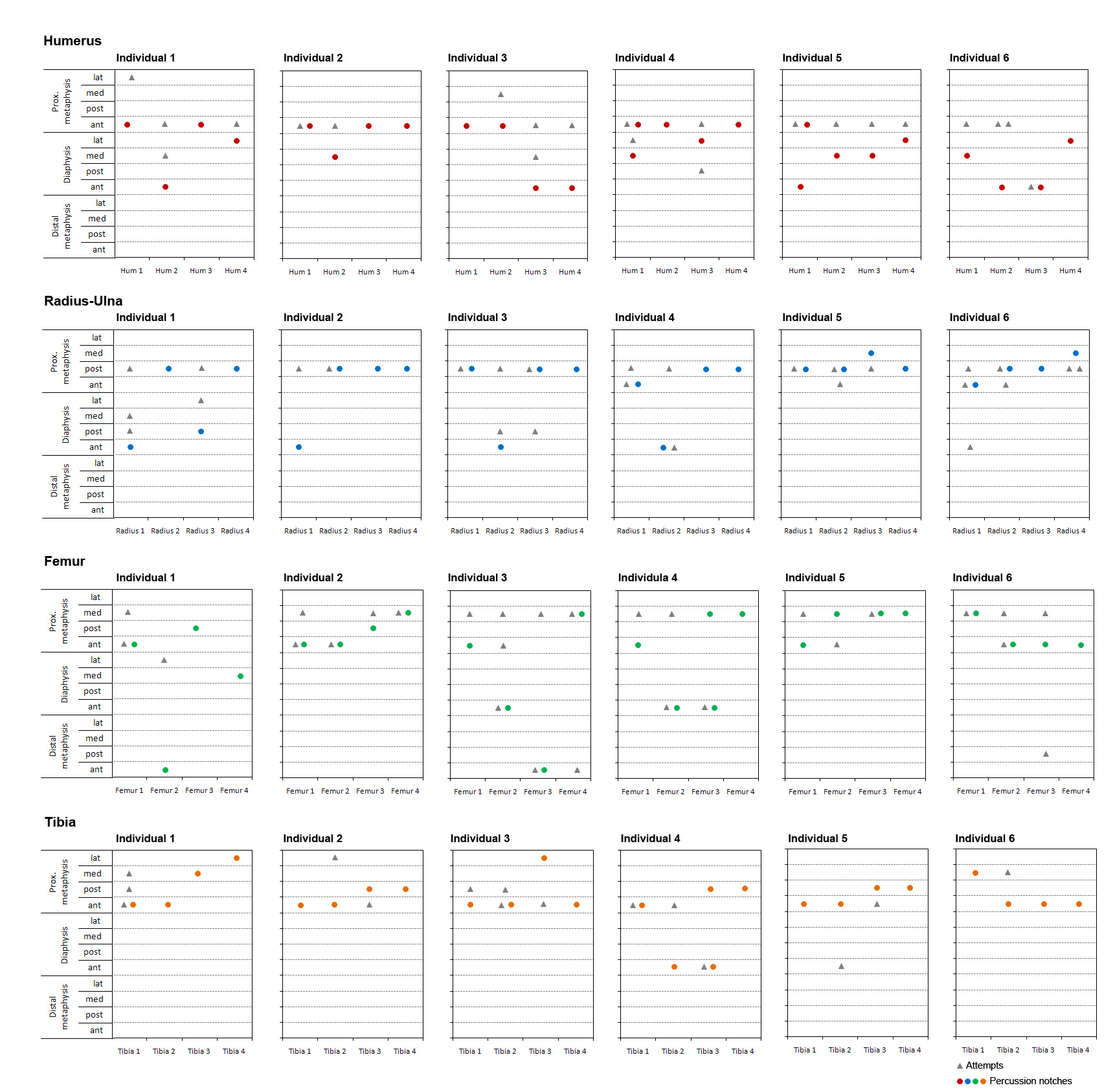
**

Figure 6. Summary of attempts and percussion notches according to skeletal elements and individuals during the development of the Experimental series 2.

*2.2 Results*

Using the EMT results detailed in the Experimental series 1, Bolomor IV long bone sample shows a distribution of notches that seems patterned (p-values: humerus >0.000; radius= 0.043; femur= 0.010 and tibia >0.000). This pattern is specifically noticeable in the humerus and tibia but barely so in the radius and femur (p-value <0.05 indicates that the observed model differs in a more patterned way from the ab-initio model). This situation is corrected when checking for the number of bone portions recovered for each of our 12 parameters, since Bolomor IV presents a sampling bias that is especially significant for radius and femur (p-values: humerus >0.000 ; radius= 0.025; femur= 0.0001 and tibia=>0.000) (see Results in Experimental series 1).

When analyzing the Experimental series 2 through EMT the results (p-value) were:

| Humerus | **0.000** |
| --- | --- |
| Radius | **0.000** |
| Femur | **0.000** |
| Tibia | **0.000** |

This shows that notches made in *all* long bones are patterned despite that none of the experimental subjects saw the others or were instructed as to a determined way of breaking the bone. This type of bone-breaking process makes individuals select the impact area by judging the ergonomics of the anvil and the bone.

When comparing the six individuals, to document if each of them produced a different pattern, the FET produced the following results:

|  | p-value |
| --- | --- |
| Humerus | 0.854 |
| Radius | 0.999 |
| Femur | 0.618 |
| Tibia | 0.425 |
|  |  |

That is no significant differences were reported in the patterns documented in each of the individual experimenters. These results show that the patterns produced by the six individuals independently were the same on all long bones, since no significant difference was detected through the FET.

Given that Bolomor IV had a large sample of notches and is presented in this work as a potential example of knowledge-mediated bone breakage, the notch distribution patterns from this archaeological level were compared to those produced in this experiment. A FET analysis yielded a significant difference between the patterns documented in the experimental series 2 and those from the site. Results of FET were:

|  | p-value |
| --- | --- |
| Humerus | **0.000** |
| Radius | **0.014** |
| Femur | **0.003** |
| Tibia | **0.000** |
|  |  |

The FET results suggest that such patterns are not the result of the main application of the percussion by batting and are very likely the result of an acquired behavioural repertoire in bone breakage.

The graphical representation of the contingency tables (via correspondence analysis) for each long meaty bone shows the same results in more detail.

Figure 7


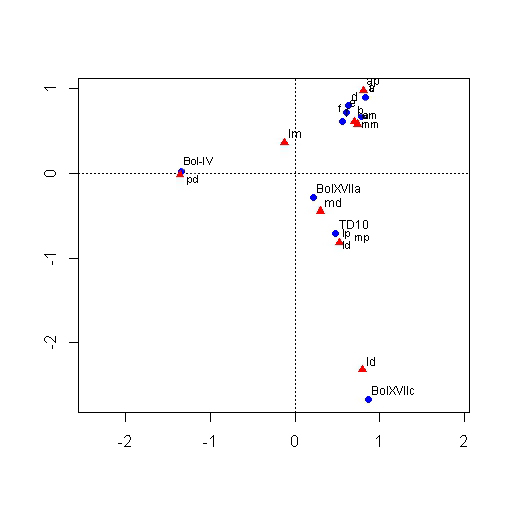


Figures show 2D solutions explaining 89.7% of the inertia with each experimenter represented by an alphabetic character. The Figure 7, corresponding to humerus, reproduces the two observations made above, namely the experimental sample is highly clustered and separated from the archaeological sites (which, are also separated among them).

Figure 8


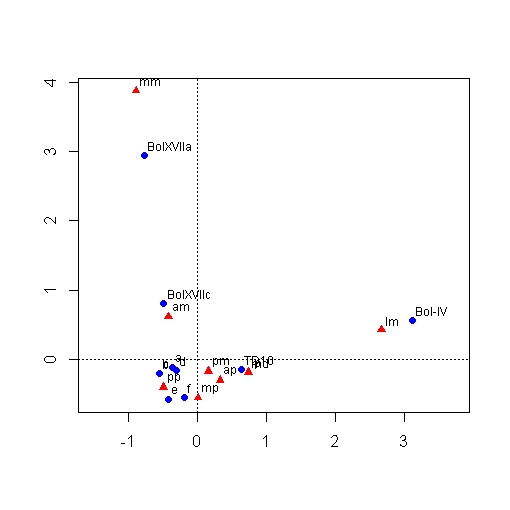


Figure 8 shows a correspondence analysis on notch location on the radii, where the experimental sample is highly clustered and separated from the archaeological cases. Bolomor XVIIc is mostly influenced by impact notches on by anterior side of mid-shaft and Bolomor IV by lateral side of mid-shaft. It also shows that all archaeological levels from Bolomor appear quite separated from the experimental sample, while TD-10-1 is closer because of the more diverse location of notches.

Figure 9


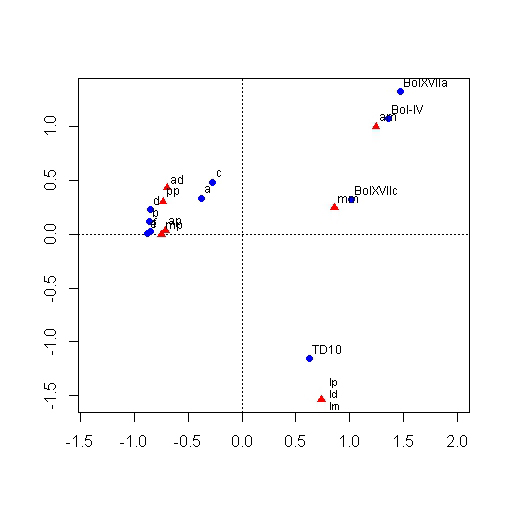


Figure 9 shows the graphical representation via correspondence analysis for femora. Following the dynamic observed on the above-mentioned skeletal elements, here the experimental sample is also highly clustered and separated from the archaeological samples. Bolomor IV is influenced by notches on the anterior side of the mid-shaft and Bolomor XVIIc on the medial side of the mid-shaft.

Figure 10


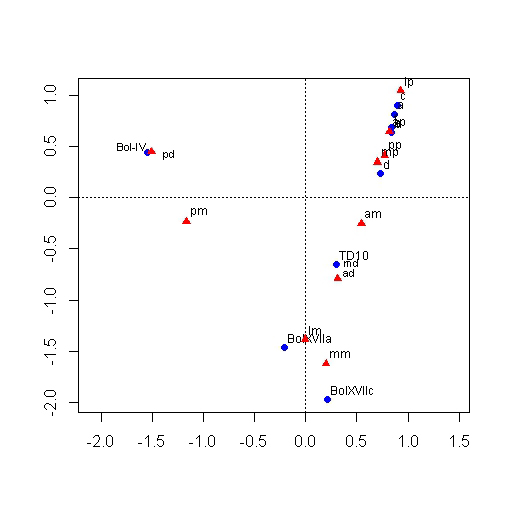


In Figure 10, all archaeological sites are separated from the experimental sample. They also appear separated among them. TD-10-1 and Bolomor XVIIa had a more diverse distribution of notches. On the contrary, Bolomor IV had a core specialized occurrence with impacts on the posterior side of the distal metadiaphysis.

*2.3 Discussion and conclusions*

Bolomor IV shows distributions statistically different from a uniformly distributed case (i.e., when EMT were applied to each bone, we obtained a p-value < 0.05 for the radius and p-values < 0.001 for the rest of cases) (see Table S2), suggesting a pattern in the selection of section and sides where impact blows have generated notches. This pattern is statistically detected on humeri and tibiae and describes counter-intuitive parameters when the FET is applied to compare archaeological and experimental samples in the Experimental series 1. Although the presence of counterblows allows us to infer the use of hammerstone percussion with anvil, we could wonder whether the use of another technique could generate similar patterns to those observed at Bolomor IV and in turn, different from those caused by hammerstone percussion.

During the Experimental series 2, the non-trained butchers generated patterned bone breakage using the percussion by batting, as documented in the fact that no significant differences were detected in the distribution of notches generated in each of the individual experimenters through the FET. This fact shows that the patterns produced by the six individuals were the same on all long bones without requiring any learning or instruction. Similar phenomena were observed by Peretto et al. [2] during the development of experiments related to this technique. These authors notice the morphology of the bones highly conditions the location of impacts and describe photographically how the subjects hold the bones by the narrower zone and hit by the wider area. The results from our series coincide with data provided by Pereto et al. [2] and allow us to back up the fact that this type of bone-breaking process makes individuals select the impact area by judging the ergonomics of bone and anvil. Given that such patterns are mainly determined by the morphological characteristics of the bone, one could argue that patterns producing notches in different bone portions from those documented in this experiment could suggest that such patterns are culturally-induced. In order to interpret whether the Bolomor IV bone breakage patterns were produced by either process, the notch distribution patterns from this archaeological level were compared to those produced in this experiment. The FET results show a significant difference between the patterns documented in the experiment and those from the site, suggesting that such patterns are not the result of the use of percussion by batting and are very likely the result of acquired knowledge in bone breakage technique.

The case of Bolomor IV alerts us about this phenomenon of counter-intuitive standardization and the possibility of identifying social learning processes from faunal remains.

**Abbreviations for Figures**

Individual characters (a-h/a-f) correspond to the individuals involved in the experimental series (each subject is represented by an alphabetic letter). Coupled characters indicate bone region -side and portion- (e.g., ap= anterior, proximal metadiaphysis; mm=medial mid-shaft; pd= posterior, distal metadiaphysis).

**References**

[1] Giusberti G, Peretto C (1991) Évidences de la fracturation intentionnelle d’ossements animaux avec moelle dans le gisement de ‘‘La Pineta’’ de Isernia (Molise), Italie. L’Anthropologie 95: 765-778.

[2] Peretto C, Anconetani P, Crovetto C, Evangelista L, Ferrari M, et al. (1996) Aproccio sperimentale alla comprensione delle attività di sussistenza condotte nel sito di Isernia La Pineta (Molise-Italia). La fratturazione intenzionale. In: Peretto C, editor. I Reperti Paleontologici del Giacimento Paleolitico di Isernia La Pineta. Isernia: Istituto Regionale per gli Studi Storici del Molise "V. Cuoco". pp. 187-452.

[3] Anconetani P (1999) L’assemblage faunique du gisement paléolithique inférieur d’Isernia-La Pineta (Molise, Italie) et l’explotation du bison. In: Brugal JP, David F, Enloe JG, Jaubert J, editors. Le Bison: Gibier et Moyen de Subsistance des Hommes du Pale´ olithique aux Pale´ oindiens des Grandes Plaines. Actes du Colloque International, Toulouse, 1995. APDCA, Antibes, pp. 105-120.

[4] Domínguez-Rodrigo M, Barba R (2005) The taphonomic relevance of the analysis of bovid long limb bone shaft features and their application to element identification: study of bone thickness and morphology of the medullary cavity. Journal of Taphonomy 3: 29-42.

1. Skeletal elements used in the experimental series 1 were obtained from butcher shops in strict accordance with Council Directive 32/2007 of 7 November on the protection of animals at the time of slaughter or killing. [↑](#footnote-ref-2)
2. Skeletal elements used in the experimental series 2 were obtained from butcher shops in strict accordance with Council Directive 32/2007 of 7 November on the protection of animals at the time of slaughter or killing. [↑](#footnote-ref-3)
